# Single-cell multi-omics analysis identifies SPP1^+^ macrophages as key drivers of ferroptosis-mediated fibrosis in ligamentum flavum hypertrophy

**DOI:** 10.1186/s40364-025-00746-6

**Published:** 2025-02-25

**Authors:** Chengshuo Fei, Yanlin Chen, Ruiqian Tan, Xinxing Yang, Guanda Wu, Chenglong Li, Jiawei Shi, Shiyong Le, Wenjie Yang, Jiajia Xu, Liang Wang, Zhongmin Zhang

**Affiliations:** 1 Division of Spine Surgery, Department of Orthopedics, Nanfang Hospital, Southern Medical University, Guangzhou, 510515 China; 2 Division of Spine Surgery, Department of Orthopaedics, The Third Affiliated Hospital, Southern Medical University, Guangzhou, 510630 China

**Keywords:** Ligamentum flavum hypertrophy, Fibrosis, Ferroptosis, Single-cell RNA sequencing, Multi-omics analysis, Macrophages.

## Abstract

**Background:**

Ligamentum flavum hypertrophy (LFH) is a primary contributor to lumbar spinal stenosis. However, a thorough understanding of the cellular and molecular mechanisms driving LFH fibrotic progression remains incomplete.

**Methods:**

Single-cell RNA sequencing (scRNA-seq) was performed to construct the single-cell map of human ligamentum flavum (LF) samples. An integrated multi-omics approach, encompassing scRNA-seq, bulk RNA sequencing (bulk RNA-seq), and Mendelian randomization (MR), was applied to conduct comprehensive functional analysis. Clinical tissue specimens and animal models were employed to further confirm the multi-omics findings.

**Results:**

ScRNA-seq provided a single-cell level view of the fibrotic microenvironment in LF, revealing significantly increased proportions of fibroblasts, myofibroblasts, and macrophages in LFH. Using transmission electron microscopy, single-cell gene set scoring, and MR analysis, ferroptosis was identified as a critical risk factor and pathway within LFH. Subcluster analysis of fibroblasts revealed functional heterogeneity among distinct subpopulations, highlighting the functional characteristics and the metabolic dynamics of fibroblast with a high ferroptosis score (High Ferro-score FB). The quantification of gene expression at single-cell level revealed that ferroptosis increased along with fibrosis in LFH specimens, a finding further validated in both human and mice tissue sections. Consistently, bulk RNA-seq confirmed increased proportions of fibroblasts and macrophages in LFH specimens, underscoring a strong correlation between these cell types through Spearman correlation analysis. Notably, subcluster analysis of the mononuclear phagocytes identified a specific subset of SPP1^+^ macrophages (SPP1^+^ Mac) enriched in LFH, which exhibited activation of fibrosis and ferroptosis-related metabolic pathways. Cell-cell communication analysis highlighted that SPP1^+^ Mac exhibited the strongest outgoing and incoming interactions among mononuclear phagocytes in the LFH microenvironment. Ligand-receptor analysis further revealed that the SPP1-CD44 axis could serve as a key mediator regulating the activity of High Ferro-score FB. Multiplex immunofluorescence confirmed substantial Collagen I deposition and reduced Ferritin Light Chain expression in regions with SPP1-CD44 co-localization in LFH specimens.

**Conclusions:**

Our findings indicated that SPP1^+^ Mac may contribute to LFH fibrosis by regulating ferroptosis in High Ferro-score FB through the SPP1-CD44 axis. This study enhances our understanding of the cellular and molecular mechanisms underlying LFH progression, potentially improving early diagnostic strategies and identifying new therapeutic targets.

**Supplementary Information:**

The online version contains supplementary material available at 10.1186/s40364-025-00746-6.

## Background

Lumbar spinal stenosis (LSS) is a common degenerative spinal disease predominantly affecting the elderly population [1]. Epidemiological studies estimate that approximately 103 million individuals globally experience LSS-related symptoms each year [2, 3], including lower back pain, radicular leg pain, and neurogenic intermittent claudication [4]. These symptoms significantly reduce the quality of life and impose a considerable socio-economic burden. The pathogenesis of LSS is multifactorial, with contributing factors including chronic degeneration of the vertebral bodies, intervertebral discs, posterior longitudinal ligament, facet joints, and ligamentum flavum (LF) [3]. Among these, ligamentum flavum hypertrophy (LFH) is widely regarded as a key etiological factor in the development of spinal canal narrowing and subsequent symptomatology [5, 6].

Histologically, the LF is a connective tissue mainly consisting of cells, fibers, and matrix. Under normal conditions, the LF is constituted of approximately 80% elastic fibers and 20% collagen fibers [6, 7]. However, in LFH, a pathological shift occurs, marked by a decrease in elastic fibers and a concomitant increase in collagen fibers [8]. The hallmark of LFH is fibrosis of the LF, which results from fibroblast activation and proliferation, along with excessive deposition of the extracellular matrix (ECM) components, predominantly collagen fibers [5]. Previous studies have shown that LFH initiates and progresses due to mechanical stress, inflammation, and oxidative stress. Despite these findings, a complete understanding of its pathogenesis remains elusive. Consequently, promising therapeutic strategies for slowing down or even reversing LFH fibrosis are scarce.

Fibroblasts and their differentiated myofibroblasts are considered the principal cellular components of LF tissue [9, 10]. Existing studies on cellular changes in LF degeneration primarily focus on myofibroblast transformation and chondroid metaplasia. However, recent investigations into LFH have broadened focus to include other cell types, such as vascular endothelial cells [11], macrophages [12], and T cells [13]. Among these cells, macrophages, as a crucial component in inflammation and injury repair, are considered as a key regulator of fibrosis initiation and progression [14]. In several fibrotic diseases, disrupted crosstalk between macrophages and fibroblasts has been observed to drive fibrosis [15]. Nevertheless, the precise role of these cellular interactions in LFH remains poorly understood. Specifically, the limited research into the cellular heterogeneity and intercellular communication in LFH, particularly at the single-cell level, has hindered our further in-depth exploration of this fibrosis mechanism. As single-cell RNA sequencing (scRNA-seq) technology evolves [16], it provides us with an approach to analyzing cellular heterogeneity in LFH, revealing the distribution and functional characteristics of specific phenotypic cells.

In most cases, uncontrolled tissue repair and wound healing, triggered by persistent damage and parenchymal cell destruction, are predisposing factors for fibrotic diseases [15]. Both cellular necrosis and programmed cell death (PCD) promote sterile inflammation, immune cell activation, and the formation of local infiltrating foci [17], all of which promote fibrosis progression. One type of PCD is ferroptosis, an iron-dependent non-apoptotic cell death [18], characterized by cysteine starvation, glutathione consumption, iron overload, and associated lipid peroxidation [19]. Recent studies have made significant strides in elucidating the role of ferroptosis in fibrotic diseases, with findings indicating that ferroptosis acts as a key driver of fibrosis in major organs such as hearts, lungs, and kidneys [20]. Therefore, targeting ferroptosis has shown emerging potential in treating multi-organ fibrosis. However, the correlation between ferroptosis and LFH remains unexplored. Thus, further research into the involvement of ferroptosis in LFH is warranted, with the aim of identifying potential diagnostic and therapeutic targets for this condition.

In this study, we employed scRNA-seq and bulk RNA sequencing (bulk RNA-seq) to conduct a multi-omic analysis of LF samples. Specifically, we generated single-cell maps of normal and degenerative LF, elaborating the cellular heterogeneity and functional alterations in LFH. Additionally, we utilized Mendelian randomization (MR) to investigate the causal relationship between iron metabolism and LFH. Notably, we identified fibroblast with a high ferroptosis score (High Ferro-score FB) in fibroblast subpopulations and determined disease-specific SPP1^+^ macrophages (SPP1^+^ Mac) in mononuclear phagocytes (MPs). Using human clinical samples and a bipedal standing (BS) mouse model [21], we validated the differential expression of ferroptosis and fibrosis markers, elucidating the potential mechanisms of interaction between SPP1^+^ Mac and High Ferro-score FB. Overall, we innovatively described the fibrotic microenvironment landscape of LFH at the single-cell level, revealing and validating the roles of ferroptosis and SPP1^+^ Mac in LFH fibrosis progression. These findings enhance our understanding of the pathogenesis and progression of LFH and provide potential clues for developing new diagnostic and therapeutic strategies.

## Methods

### Human LF tissue specimens

This study was approved by the Institutional Review Board of Nanfang Hospital of Southern Medical University (NFEC-2022-175). All research processes adhered to the ethical standards of the Institutional Review Board and the 1975 Declaration of Helsinki, as revised in 2000. Informed consent was obtained from all patients included in this study. A total of 26 patients were recruited from Nanfang Hospital, Southern Medical University (Guangzhou, China), comprising 13 with lumbar disc herniation (LDH) and 13 with LSS. Patients with spondylolisthesis, ankylosing spondylitis, rheumatoid arthritis, spinal tumors, or spinal infections were excluded. The L4/5 segment LF thickness of the patients was measured using magnetic resonance imaging (MRI). LF specimens from LDH patients with an LF thickness (≤ 3.74 mm) were categorized into the non-LFH group, while specimens from LSS patients with an LF thickness (> 3.74 mm) were categorized into the LFH group. All LF specimens were obtained from the dorsal side of the L4/5 segment during surgery. Details of the LF specimens used are provided in Additional file 1: Table S1.

### Single-cell RNA sequencing

LF tissue samples were obtained from a total of 10 donors, with 5 from the non-LFH group and 5 from the LFH group. The samples were surgically extracted and then minced and digested into single-cell suspensions using collagenase at 37 °C for scRNA-seq. BD Rhapsody™ Express single-cell analysis system (BD Biosciences, San Jose, California, USA) was used to isolate single cells and synthesize cDNA. Cells were labeled with the BD Human Single-Cell Multiplexing Kit (#633781, BD Biosciences), and cDNA libraries were established using the BD Rhapsody Whole Transcriptome Analysis (WTA) Reagent Kit (#633801, BD Biosciences). The quality of final libraries was assessed using Agilent 2100 Bioanalyzer and Qubit Fluorometer (ThermoFisher, Waltham, MA, USA) with Qubit dsDNA HS Assay Kit (#Q32854, Invitrogen, Carlsbad, CA, USA). Finally, sequencing was performed using the Illumina HiSeq X Ten sequencer (Illumina, San Diego, CA, USA) with the HiSeq X Ten Reagent Kit V2. Sequenced data from FASTQ files were analyzed utilizing the Seven Bridges platform provided by BD Biosciences to obtain raw gene expression matrices.

### Preprocessing of ScRNA-seq data

The raw gene expression matrices were loaded into R Studio (version 4.1.3) using the Seurat package (v 4.3.0) for further analysis. To filter out doublets, empty droplets, and low-quality cells, we applied the following parameters: nFeature_RNA > 200 & nFeature_RNA < 7000, nCount_RNA > 200 & nCount_RNA < 40,000. Additionally, cells with mitochondrial content (≥ 40%) and hemoglobin content (≥ 20%) were excluded during preliminary quality control. Further quality control was performed using the decontX package (v 1.0.0) to remove low-quality cells and RNA contamination, including the anomalous gene MT-RNR2. Finally, we retained 73,866 high-quality single cells for down-stream analysis.

### Integration, dimensionality reduction, clustering, and cell annotation

Transcript counts were log-normalized using the “NormalizeData” function in the Seurat package. The first 2000 variable genes were identified using the “FindVariableGenes” function, and data scaling was performed with the “ScaleData” function. We used the Harmony package (v 1.2.0) to eliminate the batch effect and integrate multiple single-cell samples. Clustering at different resolutions was examined using the clustree package (v 0.5.1), with the resolution set to 0.6. Uniform manifold approximation and projection (UMAP) was used for dimensionality reduction and display, resulting in 21 clusters. Given that myofibroblasts are key components and transformation part of LF fibroblasts, we extracted both the fibroblast and myofibroblast clusters as the fibroblast group for subclustering and reclustering, setting the resolution to 0.3. This approach identified 8 subclusters. For a second round of subclustering focused on MPs, the resolution was set to 0.5, yielding 8 additional subclusters. Differentially expressed genes (DEGs) between clusters were identified using the “FindAllMarkers” function in the Seurat package, with the log2 fold change (logFC) threshold (< 0.25), “min.pct” (0.25), and the first 50 DEGs by expression selected as marker genes. Cell types were annotated based on specific genes corresponding to different subpopulations, referencing the CellMarker database (http://xteam.xbio.top/CellMarker/), the Cell Taxonomy database (https://ngdc.cncb.ac.cn/celltaxonomy/), and various ligament-related studies. Finally, we used the scRNAtoolVis package (v 0.0.7), the ggplot2 package (v 3.4.4), and other tools to display the data.

### Single-cell functional enrichment analysis, single-cell gene set scoring and single-cell metabolism analysis

For functional enrichment analysis, we used the clusterProfiler package to perform Gene Ontology (GO) enrichment analysis on the first 50 DEGs for each fibroblast cluster. We conducted Gene Set Variation Analysis (GSVA) on all fibroblasts using the GSVA package. The reference gene sets for these analyses were obtained from the C5 ontology gene sets in the Molecular Signatures Database (MSigDB; https://www.gsea-msigdb.org). For single-cell metabolic analysis, we employed the scMetabolism package to analyze each fibroblast subgroup and used the ggradar package to visualize the relative abundance of ferroptosis-related metabolic pathways within each subgroup. Subsequently, we performed single-cell gene set scoring for all cells using the “AddModuleScore” function in the Seurat package, with reference gene sets from the C5 ontology gene sets in MSigDB. For single-cell ferroptosis scoring, we extracted ferroptosis-related genes from the “WP_FERROPTOSIS” entry in the C2 curated gene sets, classifying them into ferroptosis-promoting and ferroptosis-resisting genes based on literature (Additional file 2: Table S2). We then applied the “AddModuleScore” function to categorize all cells into “High Ferro-score cells” (cells with high expression of ferroptosis-promoting genes and low expression of ferroptosis-resisting genes), “Low Ferro-score cells” (cells with high expression of ferroptosis-resisting genes and low expression of ferroptosis-promoting genes), and “No significant cells” (cells with no differential expression of ferroptosis-promoting and ferroptosis-resisting genes). For gene set scoring of mononuclear phagocyte populations, we utilized the M1/M2 scoring gene sets proposed by Liu et al. [22]. The “FeaturePlot” function was used to map the gene expression scores of a given gene set onto each cell on the UMAP.

### Pseudotime analysis

The CytoTRACE package (v 0.3.3) was utilized to calculate CytoTRACE scores for fibroblasts, allowing for the comparison of cellular differentiation states among fibroblasts and the prediction of their relative differentiation status [23]. Next, we applied the Vector algorithm to map single-cell RNA dynamics [24], which inferred the vector of fibroblast developmental direction based on the grid distance between cells in UMAP and the CytoTRACE-predicted starting cells. To identify the differentiation trajectory of the fibroblast population, the Monocle2 package (v 2.2.2) was used to compute differentiation trajectories and to perform branch expression analysis modeling for gene analysis related to branch fate [25]. Additionally, the “plot_genes_branched_heatmap” function was used to generate a heatmap of gene clustering. Lastly, pseudotime expression trends of ferroptosis-promoting and ferroptosis-resisting gene sets were displayed with the ggplot2 package.

### Bulk RNA sequencing and transcriptome analysis

LF tissue samples from 6 donors (3 from the non-LFH group and 3 from the LFH group) were surgically collected for transcriptome sequencing (GSEzzm, this data has not yet been uploaded to the public database). We retrieved the microarray dataset GSE113212 from the Gene Expression Omnibus (GEO) database, which includes 4 hypertrophied LF samples from elderly individuals and 4 non-hypertrophied LF samples from younger individuals. To increase the sample size for RNA-seq data, we combined transcriptome sequencing data with microarray datasets using the “Combine” function from the dplyr package (v 1.3.9000) and corrected batch effects using the “Combat” function from the sva package (v 3.42.0). Principal component analysis (PCA) was then performed with the ggord package (v 1.1.8) to assess the quality of the merged and normalized data. Differential expression analysis was conducted using the limma package, with an FC threshold of 1.5 and a *p*-value of 0.05 to identify DEGs. To identify significantly enriched biological processes between the non-LFH and LFH groups, we used the C5 ontology gene sets (c5.all.v51.symbols.gmt) from MSigDB as the reference gene set. Then we performed Gene Set Enrichment Analysis (GSEA) with the clusterProfiler package and displayed the results using the enrichplot package (v 1.14.2).

### Deconvolution analysis

Using the BayesPrism package (v 2.1.2) [26], we leveraged scRNA-seq data of LFH as prior information to perform cell type and gene expression deconvolution on the merged bulk RNA-seq data. This approach allowed us to infer the composition and expression levels of fibroblasts and immune cells in LF tissue. Spearman correlation analysis was conducted to compare correlations between fibroblasts and other immune cells in the deconvolution data, with heatmaps used for visualization.

### Cell-cell communication analysis

To analyze potential cell-cell interactions, we created objects separately for LFH and non-LFH group samples from Seurat objects based on CellChat (v 2.2.1) pipeline [27]. We utilized the CellChatDB.human dataset from the CellChatDB database, selecting “Secreted Signaling”, “ECM-Receptor”, “No-protein Signaling” and “Cell-Cell Contact” as communicating ways. Then, we identified highly expressed ligands and receptors and assessed their interactions. Communication probabilities between cell subpopulations were calculated using the “computeCommunProb” function with the “truncatedMean” method, which allowed us to infer the cell communication network. Subsequently, a comparative analysis of CellChat objects for LFH and non-LFH groups was performed, examining interaction strength between cells in a two-dimensional space to identify significant changes in signaling or receiving signals across different groups. Differential expression analysis was conducted to identify upregulated and downregulated L-R pairs. Then, interaction quantities between cell types were visualized using bubble plots and chord diagrams to illustrate cell-cell communication networks and highlight significant interactions between groups.

### Two-sample Mendelian randomization analysis

Under the guidance of the STROBE-MR statement [28], we designed an MR study to investigate the causal relationship between ferroptosis and LFH. Exposure data were obtained from the Disorders of Iron Metabolism (finn-b-E4_IRON_MET) dataset in the Finnish Genetics. The total sample size was 197,405 individuals, all of European descent, with 146 diagnosed with iron metabolism disorders and 197,259 serving as controls. A total of 16,380,377 single nucleotide polymorphisms (SNPs) were included. Since LFH data were not available in the genome-wide association study (GWAS) database, we used spinal canal stenosis (SCS) data instead. The outcome dataset included a total of 454,787 individuals with SCS (ebi-a-GCST90018922) from the European Bioinformatics Institute database, all of European descent. Among them, 9,660 individuals had SCS, while 445,127 served as controls, with a total of 24,182,979 SNPs analyzed. We selected SNPs that are strongly associated with the exposure factor (*p*-value < 5 × 10 − 8) as instrumental variables and further removed SNPs with linkage disequilibrium and weak instrumental variables. We then employed the inverse-variance weighted (IVW) approach from the TwoSampleMR package (v 0.5.7) as the primary analysis method, with weighted mode and MR-Egger serving as complementary methods. To confirm the reliability of the MR results, we conducted sensitivity analyses, including tests for horizontal pleiotropy [29], heterogeneity [30], and leave-one-out analysis.

### Transmission electron microscopy

LF samples were fixed overnight in a 2.5% glutaraldehyde solution. After dehydration, embedding, and polymerization, the samples were sectioned into ultrathin slices approximately 100 nm thick using an ultramicrotome. Subsequently, the sections were stained with 2% uranyl acetate saturated alcohol solution and lead citrate. Transmission electron microscopy (TEM) images were taken with a Tecnai G2 Spirit transmission electron microscope (FEI Company, Czech Republic).

### Mouse experiments

All animal experiments were approved by the Animal Experiment Ethics Committee of Nanfang Hospital of Southern Medical University (Approval No.nfyy-2021-1021) and followed the National Institutes of Health guide for the care and use of Laboratory animals (NIH Publications No. 8023, revised 1978). Twelve 8-week-old male C57BL/6 mice were obtained from the Laboratory Animal Research Center at Southern Medical University. The LFH mouse model was developed based on previous research findings [21, 31]. The mice were randomly assigned to either the control group (*n* = 6) or the BS group (*n* = 6). During the 12-week modeling period, BS group mice were housed in individual modeling devices with water (5 mm depth) at the bottom to induce a BS posture through their aversion to water. Control group mice were placed in similar devices without water, while all other housing conditions were kept identical. Mice in both groups were exposed to the modeling devices for 8 h daily, with 2-hour intervals allowed for free feeding and drinking. After 12 weeks, all mice were euthanized, and complete L5/6 spinal specimens were collected for Masson, Elastica van gieson (EVG) and IHC staining.

### Detection of ferrous ions

The total level of ferrous ions in LF tissue samples was measured with the Ferrous Ion Content Assay Kit (BC5415, Solarbio, Beijing, China) according to the manufacturer’s protocol. Specifically, the LF tissues collected during surgery were weighed, homogenized in Reagent I in an ice bath, and centrifuged at 10,000 × g and 4 ℃ for 10 min. Next, 200µL of samples were fully mixed with 100µL of Reagent II, and incubated at 37℃ for 10 min. Subsequently, 100µL of CHCL3 was added to the samples, followed by shaking for 5 min and centrifugation at 12,000 × g at room temperature. Finally, 200µL of the processed tissue samples and diluted standard samples were added to 96-well plates, and the absorbance was measured using a microplate reader at 593 nm. The ferrous ion concentration in each sample was calculated based on the standard curve.

### Histological studies and immunohistochemical staining

Human LF specimens and mouse lumbar spinal specimens were fixed in 4% paraformaldehyde for 48 h and then decalcified with EDTA solution (SH5546, Sihe Biotechnology, Guangzhou, China) for 1 month. After gradient alcohol dehydration, the specimens were paraffin-embedded and sectioned into 4 μm slices. Histologically, the tissue slices were placed in a 65 °C oven for 2 h, then dewaxed and rehydrated. The slices were stained using the EVG staining kit (B1053, Baiqiandu, Wuhan, China) and the Masson’s Trichrome Stain Kit (B1011, Baiqiandu, Wuhan, China), according to the kit instructions. For IHC staining, the tissue slices were heated in a 65 °C oven for 3 h, followed by dewaxing and rehydration. Antigen retrieval was performed using a microwave, and the slices were then cooled to room temperature for 2 h. The slices were treated with 3% hydrogen peroxide in the dark for 15 min, blocked with ready-to-use goat serum (AR0009, Boster, China) for 1 h to prevent nonspecific binding, and incubated overnight at 4 °C with specific antibodies against GPX4 (#DF6701, 1:100, Affinity), FTL (#DF6604, 1:100, Affinity), ACSL4 (#DF12141, 1:100, Affinity), COL1 (#AF7001, 1:100, Affinity), α-SMA (#AF1032, 1:100, Affinity), and FN1 (#AF5335, 1:100, Affinity). The next day, the slices were incubated with goat anti-rabbit IgG (H + L) horseradish peroxidase (HRP) secondary antibody (BF03008X, Biodragon) at room temperature for 2 h. IHC staining was then carried out using the DAB kit (Service-Bio, Shanghai, China), with hematoxylin used for nuclei staining. Subsequently, we scanned stained slices and collected images using a high-resolution digital slide scanner (Pannoramic SCAN, 3DHISTECH, Hungary). Lastly, ImageJ software (NIH, USA) was used to measure the ratio of elastic fibers to collagen fibers in histological studies and the proportion of positive staining cells in IHC, followed by quantitative analysis.

### Western blotting

LF specimens were collected during surgery, washed with PBS to remove excess fat and bone tissue, rapidly frozen in liquid nitrogen, and stored at -80 °C. Total protein was extracted from human LF specimens using RIPA lysis buffer (Santa Cruz). Then, protein concentration was determined with a BCA protein assay kit (Pierce). Proteins were separated by SDS-PAGE after denaturation in a 100 °C metal bath, with prestained protein ladder provided by ThermoFisher (#26616, ThermoFisher). Following separation, proteins were transferred to a PVDF membrane (Roche Applied Science, Indianapolis, IN, USA) by Western blotting. Blocking was performed at room temperature for 15–20 min using a rapid blocking buffer (PS108, Epizyme Biomedical Technology, Shanghai, China). The membrane was then incubated overnight at 4 °C with specific antibodies against GPX4 (#DF6701, 1:1000, Affinity), FTL (#DF6604, 1:1000, Affinity), ACSL4 (#DF12141, 1:1000, Affinity), COL1 (#T61022, 1:1000, Abmart), α-SMA (#AF1032, 1:1000, Affinity), and FN1 (#MN50073, 1:1000, Abmart), with GAPDH (1:5000, AP0063, Bioworld) as loading control. After washing with TBS-T, the membrane was incubated at room temperature for 1 h with IRDye 680RD goat anti-mouse secondary (#926-68070, 1:10,000, LI-COR Biosciences) and IRDye 800CW goat anti-rabbit secondary (#926-32211, 1:10,000, LI-COR Biosciences). The membrane was washed three times with TBS-T and imaged on Odyssey Dual-color infrared laser imaging system (LI-COR). Western blot analysis were done using Image Studio Ver 5.2 software.

### Multiplex immunofluorescence staining

Multiplex immunofluorescence staining was performed using the tyramide signal amplification (TSA)-based system (Baiqiandu, Wuhan, China). Specifically, we deparaffinized the LF tissue paraffin sections, rehydrated them, and performed heat-induced antigen retrieval. Subsequently, sections were incubated in the 3% hydrogen peroxide solution at room temperature in the dark for 25 min, and blocked with 10% goat serum at room temperature for 30 min. To present cell communication, sequentially, sections were incubated overnight at 4 °C with different primary antibodies on the same tissue slide. The corresponding polymer HRP-conjugated secondary antibodies (anti-goat, anti-rabbit, anti-mouse, anti-rat IgG) were applied to the sections, and those were incubated at room temperature in the dark for 50 min. Next, the sections were incubated with TSA reagents mixed with fluorescent dyes for 20 min. After each TSA reagent process, we performed microwave antigen retrieval and reblocking before incubating with the next round of antibodies and dyes. The primary antibodies used were SPP1(#PB0589, 1:200, Boster), CD44 (#15675-1-AP, 1:1000, proteintech), COL1 (#AF7001, 1:200, Affinity), and FTL (#DF6604, 1:200, Affinity), while the corresponding dyes were tyramide CY3, tyramide-488, tyramide-594, and tyramide-450a. Finally, the cell nuclei were stained with DAPI and the high-resolution digital slide scanning was performed utilizing a Pannoramic SCAN (3DHISTECH CaseViewer, Hungary) to capture multiple IHC images.

### Statistical analysis

All statistical analyses were conducted using R software (RStudio version 4.1.3), SPSS 20.0 software (SPSS Inc., Chicago, USA), and GraphPad Prism 9.0.0 (GraphPad Software, La Jolla, USA). Data are presented as mean ± standard deviation. The independent samples *t*-test (Students’ *t*-test) and Wilcoxon test were applied to compare the sample means of the two groups. For the experimental data, the unpaired T-test was selected to compare the two sample means, and Welch’s ANOVA was required for inconsistent variances. A *p*-value (*p* < 0.05) was considered statistically significant. ‘ns’ represent *p* > 0.05, *p < 0.05, **p < 0.01, ***p < 0.001, ****p < 0.0001.

## Results

### Comprehensive scRNA-seq analysis unveils the cellular landscape in human LF tissue

**Fig. 1:**
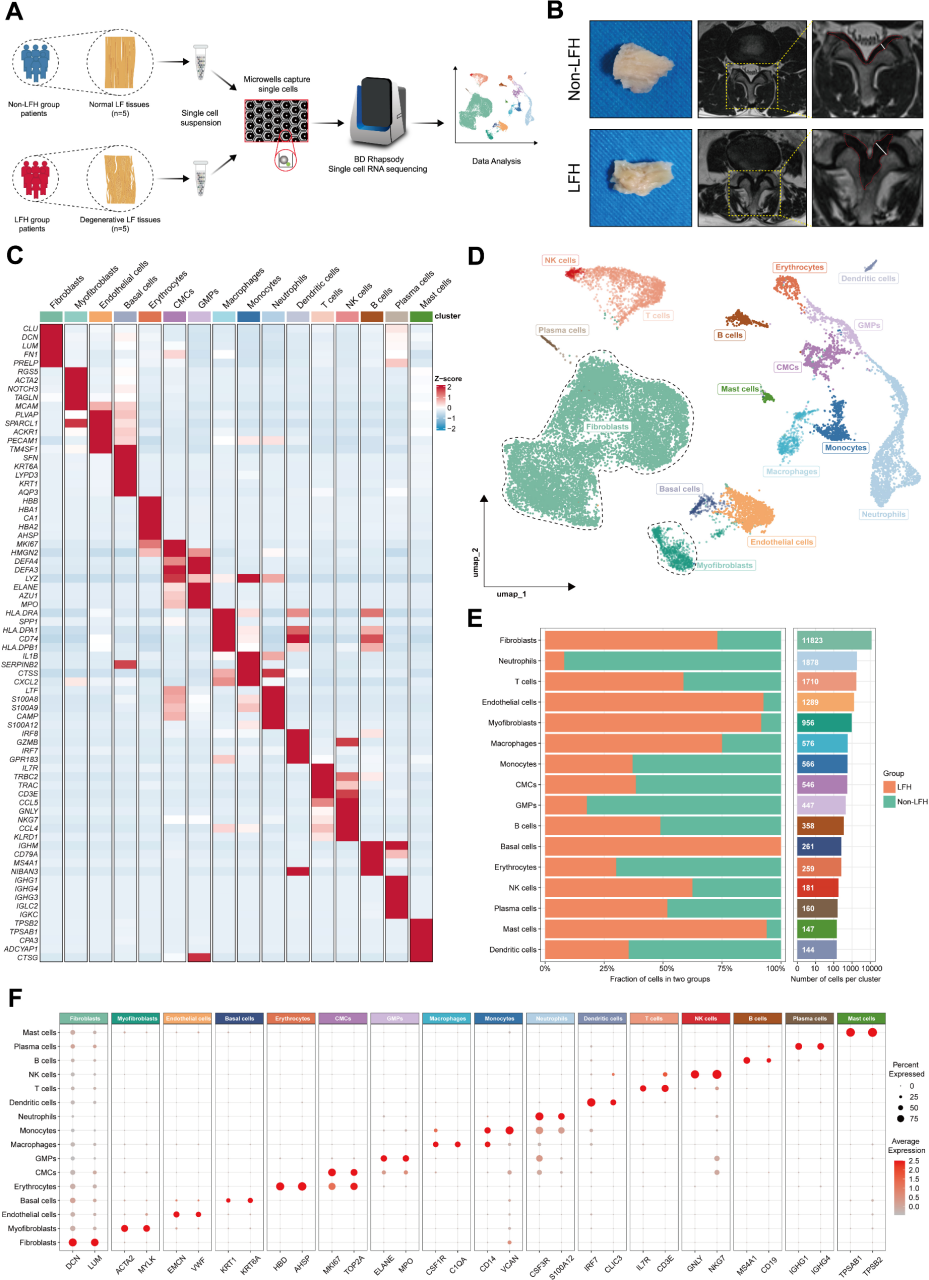
Comprehensive scRNA-seq analysis unveils the cellular landscape in human LF tissue. **(A)** Workflow of scRNA-seq, including sample collection, digestion, sequencing, and analysis. **(B)** Representative macroscopic images and lumbar MRI images from the non-LFH and LFH groups. **(C)** Heatmap showing the expression of the first five DEGs for different lineages. Red indicates the high expression, blue indicates the low expression. **(D)** UMAP visualization of 21,301 cells from the human LF tissue, dyed according to cell types. Fibroblasts and myofibroblasts are circled by the black dotted line. **(E)** Cell proportion plot showing the proportions of 16 cell groups in the non-LFH and LFH groups. The bar chart on the right shows their cell numbers. **(F)** Dot plot showing the expression of characteristic genes of different cell types. The size of the dot represents the proportion of expressing cells; the colors show the normalized gene expression intensity

To investigate the cellular heterogeneity of human LF tissue and the pathogenesis of LFH at the single-cell level, we performed scRNA-seq on LF samples obtained from 10 donors, comprising 5 non-LFH and 5 LFH samples. The complete workflow is illustrated in Fig. 1A. All LF samples were classified based on clinical information, lumbar MRI images, and intraoperative observations. Preoperative MRI measurements confirmed that LF thickness was less than or equal to 3.74 mm in the non-LFH group and more than 3.74 mm in the LFH group (Fig. 1B) [32]. The clinical information of donors for scRNA-seq is included in Additional file 3: Table S3. The Morphological examination showed that normal LF samples were light yellow with a striped pattern, clear and flat in structure, and had an elastic texture. In contrast, hypertrophic LF samples appeared darker and exhibited disorganized structure, swelling, and a firmer texture (Fig. 1B).

To remove low-quality cells and control the contamination of RNA, we preprocessed the dataset and performed data quality control, retaining a total of 21,301 cells for subsequent analysis. Next, we processed the scRNA-seq data using the Seurat standard pipeline and applied unsupervised UMAP clustering, which revealed 21 clusters, as shown in Additional file 7: Fig. S1. Then we identified these marker genes for each cell cluster (Additional file 4: Table S4) and respectively annotated clusters using classic lineage markers. The first five DEGs expressed in each cell type were displayed with a heatmap (Fig. 1C). With UMAP, we mapped the single-cell landscape of human LF tissue (Fig. 1D) and identified 16 major cell types, including Fibroblasts (*n* = 11823) marked by *DCN* and *FN1*; Myofibroblasts (*n* = 956) identified by *ACTA2* and *MYLK*; Endothelial cells (*n* = 1289) expressing *VWF* and *EMCN*; Basal cells (*n* = 261) marked by *KRT1* and *KRT6A*; Erythrocytes (*n* = 259) marked by *HBD* and *AHSP*; Cycling myeloid cells (CMCs, *n* = 546) marked by *MKI67*, *TOP2A*; Granulocyte monocyte progenitors (GMPs, *n* = 447) marked by *AZU1*, *ELANE*; Macrophages (*n* = 576) marked by *C1QA* and *CSF1R*, Monocytes (*n* = 566) marked by *CD14* and *VCAN*; Neutrophils (*n* = 1878) marked by *CSF3R* and *S100A12*; Dendritic cells (*n* = 144) marked by *IRF7* and *CLIC3*; T cells (*n* = 1710) marked by *IL7R* and *CD3E*; NK cells (*n* = 181) marked by *GNLY* and *NKG7*; B cells (*n* = 358) marked by *MS4A1* and *CD19*; Plasma cells (*n* = 160) marked by *IGHG1* and *IGHG4*; Mast cells (*n* = 147) marked by *TPSAB1* and *TPSB2*. The characteristic genes of each cell cluster are displayed using a Dot plot (Fig. 1F).

Previous studies have identified fibroblasts as the predominant cell type in human LF tissue [5, 33]. During the hypertrophic degeneration of LF, fibroblasts can undergo extensive proliferation and secrete large amounts of collagen, transforming into myofibroblasts characterized by increased expression of α-SMA protein encoded by *ACTA2* [34]. Additionally, macrophage infiltration is another feature of LFH progression, playing a critical role in the regulation of fibrosis. Comparing cell proportions between LF samples from the non-LFH and LFH groups, we found significant differences in the relative proportions of cell lineages between the two groups (Fig. 1E). Fibroblasts remained the primary cell type in LF samples from both groups, with notably increased proportions of fibroblasts and myofibroblasts observed in the LFH group compared to the non-LFH group. These findings indicated that our scRNA-seq results were consistent with previous studies. In addition, the proportions of endothelial cells, basal cells, macrophages, and mast cells were significantly higher in the LFH group. Therefore, we speculated that these cell types may play important roles in the repair of damaged LF and the progression of fibrosis.

In summary, these data described the fibrotic microenvironment landscape of human LF tissue, highlighting proportional alterations in fibroblasts, as well as other stromal and immune populations, during the progression of LFH.

### Ferroptosis may contribute as a potential risk factor for LFH

**Fig. 2:**
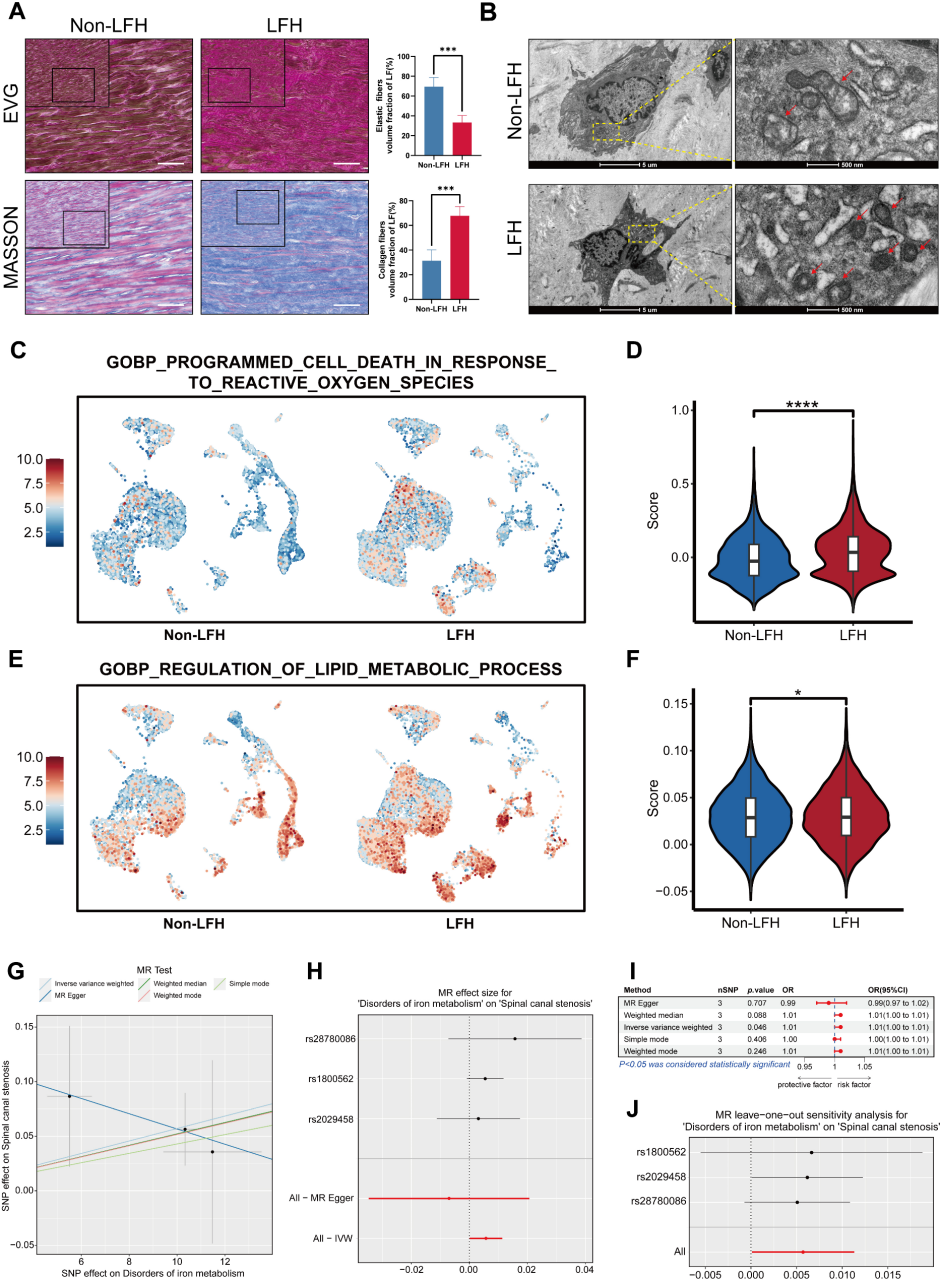
Ferroptosis may contribute as a potential risk factor for LFH. **(A)** Representative histopathological findings of LF tissue from the non-LFH and LFH groups (*n* = 5). In EVG staining, collagen fibers manifested red, and elastic fibers manifested black. In Masson’s trichrome staining, collagen fibers were stained blue, and elastic fibers were stained red. The accompanying bar graph presents the volume fractions (%) of elastic and collagen fibers in LF tissue as mean ± SD, with ***p < 0.001. Scale bar, 50 μm. **(B)** Representative TEM images of LF tissue from the non-LFH and LFH groups (*n* = 2). Scale bar: left, 5 μm; right, 500 nm. The red arrows indicate shrunken mitochondria. (**C**, **E**) UMAP plots illustrating the scoring results of the “PROGRAMMED CELL DEATH IN RESPONSE TO REACTIVE OXYGEN SPECIES” and the “REGULATION OF LIPID METABOLIC PROCESS” gene sets in scRNA-seq of the non-LFH group and the LFH group, with red indicating high scores and blue indicating low scores. (**D**, **F**) Bar graphs showing significant differences in gene set scores between the two groups (*p < 0.05,****p < 0.0001). (G) Scatter plot presenting the correlation between iron metabolism disorder and SCS across different MR methods. The x-axis represents the effect of individual SNPs on iron metabolism disorder, while the y-axis represents their effect on SCS. The slope of the line represents the causal effect for each method. (H) Forest plot demonstrating the causal effect of ferroptosis on SCS. Black dots represent the estimated effect of individual SNPs on SCS risk due to iron metabolism disorder, while red dots represent the overall effect estimated using MR-Egger and IVW methods. (I) Five methods were used in the forest plot to visualize the causal effects of iron metabolism disorders on SCS. (J) Leave-one-out sensitivity analysis: black dots represent the estimated causal effect of ferroptosis on LSS after removing each SNP, while red dots indicate the overall estimated causal effect on LSS after filtering SNPs. The x-axis shows the estimated effect of iron metabolism disorder on SCS after sequential removal of each SNP, and the y-axis shows all sequentially removed SNPs

To validate the scRNA-seq-derived samples pathologically, we performed histological staining and electron microscopy on LF tissue from both non-LFH and LFH groups. EVG and Masson staining results (Fig. 2A) showed that LF samples from the non-LFH group contained a large amount of regularly arranged elastic fibers and a small amount of collagen fibers. In contrast, LF samples from the LFH group comprised extensive proliferation of collagen fibers, which infiltrated the gaps formed by the tearing and loss of elastic fibers. Combined with the quantitative analysis, the results indicated a significant increase in collagen fibers and a notable decrease in elastic fibers in the LFH group compared to the non-LFH group. These findings suggested that LF samples from the LFH group underwent more severe degeneration and fibrosis, whereas LF tissue from the non-LFH group remained relatively normal, without significant fibrosis.

We further utilized TEM to examine LF specimens from the non-LFH and LFH groups. Compared to normal LF specimens, degenerated LF specimens from the LFH group showed mitochondrial shrinkage, increased membrane density, and reduced or absent cristae, which are characteristic features of ferroptosis (Fig. 2B). In addition, LFH tissues exhibited a higher level of ferrous ion content than non-LFH samples (Additional file 7: Fig. S2). Based on these physiological alterations in LF tissue and the characteristics of ferroptosis, we speculated that LFH is more prone to ferroptosis. It has been reported that the main manifestation of ferroptosis is lipid peroxidation caused by excessive accumulation of reactive oxygen species (ROS) in cells and an imbalance of lipid metabolism [18, 35]. With subsequent gene set scores in scRNA-seq, we found that ROS scores (Fig. 2C-D) and lipid metabolism scores (Fig. 2E-F) were significantly higher in the LFH group than in the non-LFH group. This finding indicated that ferroptosis may occur during LFH progression, characterized by increased ROS and disrupted lipid metabolism.

Based on these inferences, we sought to investigate the causal relationship between iron metabolism and the risk of LFH. Since the GWAS database does not have a specific cohort for LFH, we used “Spinal canal stenosis” as the outcome and “Disorders of iron metabolism” as the exposure factor to perform an MR analysis. The MR analysis results showed IVW (OR = 1.01, 95% CI = 1.00–1.01, *P* = 0.046), MR-Egger (OR = 0.99, 95%CI = 0.97–1.02, *P* = 0.707), and weighted mode (OR = 1.01, 95%CI = 1.00–1.01, *P* = 0.256), suggesting a positive correlation between disorders of iron metabolism and SCS (Fig. 2G-I). Subsequently, we assessed the reliability of these results through the sensitivity analysis. The IVW analysis indicated no heterogeneity (Q = 0.864, *P* = 0.649), and the MR-Egger analysis showed no horizontal pleiotropy (Egger intercept = 0.127, *P* = 0.527) (Additional file 5: Table S5). Lastly, a leave-one-out sensitivity analysis, performed by sequentially removing each SNP, demonstrated that the causal effect of iron metabolism disorder on SCS remained relatively stable (Fig. 2J). Overall, our findings reveal increased evidence of ferroptosis in degenerated LF tissue, as observed through TEM. Additionally, single-cell gene set scoring and MR analysis suggest that ferroptosis may function as a risk factor contributing to the development of LFH.

### Definition and functional distinctions of fibroblast subpopulations highlight the close association between ferroptosis and LF fibrosis

**Fig. 3:**
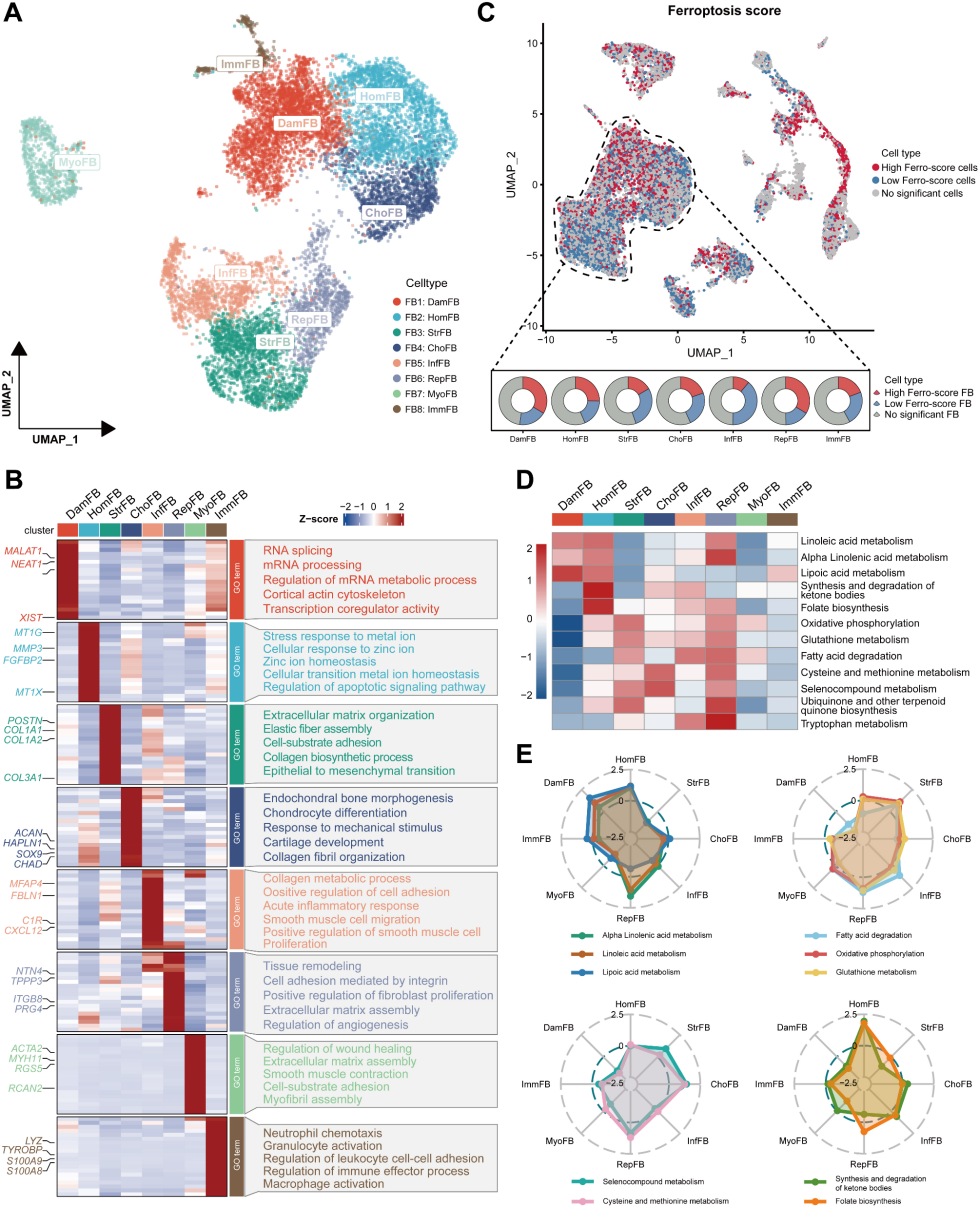
Definition and functional distinctions of fibroblast subpopulations highlight close association between ferroptosis and LF fibrosis. **(A)** UMAP visualization of fibroblast subclusters in human LF tissue, dyed according to the cell types. **(B)** Heatmap displaying the specific markers for each fibroblast cluster. Red indicates the high expression, and blue indicates the low expression. The box on the right presents the GO enrichment analysis results for the first 50 DEGs expressed in each fibroblast cluster. **(C)** UMAP plot (top) showing the ferroptosis scores of all cells, where red indicates High Ferro-score cells, blue indicates Low Ferro-score cells, and gray indicates no significant cells. The pie chart (bottom) showing the proportion of High Ferro-score FB, Low Ferro-score FB, and No significant FB in each fibroblast subpopulation. Heatmap **(D)** and radar chart **(E)** presenting significant changes in metabolic pathways related to iron and lipid metabolism across different fibroblast subpopulation. In heatmap, red indicates high activity and blue indicates low activity

Fibroblasts and myofibroblasts are the main components and effector cells in LF tissue. Then we extracted them for further clustering and functional analyses. Various markers were used to categorize fibroblasts and myofibroblasts into eight distinct subpopulations, labeled from FB1 to FB8 (Fig. 3A).

We performed GO analysis on the specific DEGs of each fibroblast subpopulation to identify their functional differences. And the results are visualized by the heatmap (Fig. 3B). FB1, designated as Damaged Fibroblast (DamFB), was enriched in GO terms such as “RNA splicing” and “mRNA processing”. And it uniquely expresses long non-coding RNAs *NEAT1*, *MALAT1* [36], and *XIST*, which are associated with aging and frailty. Compared to other fibroblasts, FB1 shows lower expressions of most other genes, indicating that it may represent functionally impaired, frailty-specific fibroblasts. FB2, characterized by the expression of metallothionein family genes such as *MT1G* and *MT1X*, is defined as Homeostasis-associated Fibroblast (HomFB). This subpopulation is associated with homeostasis and defense, consistent with previous reports on the human anterior cruciate ligament [37]. GO enrichment analysis showed that HomFB is strongly linked to cellular transition metal ion homeostasis and stress responses to metal ions, suggesting a potential role in metal ion regulation. FB3, designated as Structural Fibroblast (StrFB), is characterized by high expression of genes related to ECM components, collagen fiber and elastic fiber formation, such as *COL1A1*, *COL3A1*, and *POSTN*. It was enriched in GO terms like “extracellular matrix organization”, “elastic fiber assembly” and “collagen biosynthetic process”, indicating its role in structural support. FB4, labeled as Chondrogenic Fibroblast (ChoFB), was enriched in cartilage-related biological processes and showed high expression of cartilage-associated genes, including *ACAN*, *HAPLN1*, *SOX9*, and *CHAD*, identifying this group as chondrogenic-like fibroblasts. FB5, designated as Inflammation-related Fibroblast (InfFB), was characterized by high expression of inflammation-related genes such as *C1R* and *CXCL12*, along with ECM-related genes like *MFAP4* and *FBLN1*. GO analysis revealed enrichment in terms such as “acute inflammatory response” and “collagen metabolic process”. FB6, labeled as Repair-related Fibroblast (RepFB), specifically expressed genes associated with repair and cell proliferation, including *TPPP3* [38], *PRG4* [39], integrin-associated *ITGB8*, and angiogenesis-associated *NTN4* [40]. Enrichment analysis suggested strong associations with tissue remodeling, integrin signaling, cell adhesion, and cell proliferation, indicating its involvement in injury repair and regeneration. FB7, designated as Myofibroblast (MyoFB), corresponded to the myofibroblast population, marked by *ACTA2* and *RGS5*. Lastly, FB8, labeled as Immune-related Fibroblast (ImmFB), was primarily involved in processes such as “macrophage activation”, “elastic fiber assembly” and “collagen biosynthetic process”. This subpopulation may play a role in modulating immune cell migration and activity, potentially interacting with immune cell signals in the LF tissue.

To validate the accuracy of the fibroblast subpopulation definitions and their functional heterogeneity, we conducted a GSVA on all fibroblast subpopulations (Additional file 7: Fig. S3). The results indicated that StrFB, InfFB, RepFB, and MyoFB are enriched in biological processes associated with ECM remodeling and collagen production, with StrFB showing the highest GSVA score. This suggested that these fibroblast subpopulation are key effector cells in the LF tissue. In contrast, DamFB exhibited the lowest GSVA scores for these biological processes, reinforcing our inference that this frailty-specific fibroblast subpopulation is functionally impaired. Interestingly, DamFB also had low GSVA scores in ferroptosis-related metabolic processes, including “GLUTATHIONE PEROXIDASE ACTIVITY” and “GLUTAMINE TRANSPORT”. Additionally, in exploring the flux of fibroblast subpopulations between the non-LFH and LFH groups, we found that the proportion of DamFB significantly increased in hypertrophic LF samples, making it the most prevalent fibroblast cluster in the LFH group (Additional file 7: Fig. S4). These findings suggested that the susceptibility and functional impairment of DamFB may be closely related to ferroptosis in the LFH fibrosis process.

Next, we performed ferroptosis scoring for all cells. Based on the expression levels of ferroptosis-promoting and ferroptosis-resistant genes (Additional file 2: Table S2), we classified fibroblasts (excluding myofibroblasts) into three categories (Fig. 3C), including High Ferro-score FB, fibroblast with a low ferroptosis score (Low Ferro-score FB), and fibroblast with a no significant ferroptosis score (No significant FB). The pie chart revealed that DamFB and RepFB had the highest proportions of High Ferro-score FB, while StrFB and InfFB had the lowest proportions of Low Ferro-score FB cells (Fig. 3C). This indicated that the functional impairment and vulnerability of DamFB may be attributed to its high content of ferroptotic fibroblasts.

Previous studies have shown that ferroptosis is primarily characterized by decreased levels of glutathione metabolism and increased activity in lipid metabolism pathways such as alpha-linolenic acid metabolism [41, 42]. Therefore, we performed a detailed metabolic pathway analysis on all fibroblast subpopulations (Fig. 3D). The results indicated that DamFB, HomFB, and RepFB (with DamFB showing the highest pathway activity) are primarily enriched in metabolic pathways associated with ferroptosis-promoting, including alpha-linolenic acid metabolism, linoleic acid metabolism, and lipoic acid metabolism. However, StrFB exhibited the lowest activity in these ferroptosis-related pathways (Fig. 3D). In contrast, among metabolic pathways that protect against ferroptosis, such as glutathione metabolism, fatty acid degradation and oxidative phosphorylation, DamFB showed the lowest pathway activity, while StrFB demonstrated the highest activity (Fig. 3D). These findings showed that DamFB, which is functionally impaired and frail, has more active metabolic pathways promoting ferroptosis, leading to higher ferroptosis levels. However, functionally intact StrFB has lower ferroptosis levels and potentially greater resistance to ferroptosis. Additionally, the radar chart results (Fig. 3E) indicated that HomFB, involved in metal ion defense and regulation, may have the capacity to modulate ferroptosis-related metabolic pathways. The high metabolic rate of RepFB in all metabolic pathways indicated that it may be in a transitional phase during the repair and regeneration of LFH lesions. Furthermore, ChoFB was notably active in selenocompound metabolism and cysteine and methionine metabolism (Fig. 3E), while HomFB showed higher pathway activity in the synthesis and degradation of ketone bodies and folate biosynthesis. These findings demonstrate that the metabolic pathways are closely associated with the potential functions of these fibroblast subpopulations. In conclusion, these analyses advances our understanding of the heterogeneity and functional differences among fibroblast subpopulations in LFH, highlighting the close association between ferroptosis and these functional variations.

### Pseudotime analysis reveals the cell development trajectories of fibroblast subpopulations in LFH

**Fig. 4:**
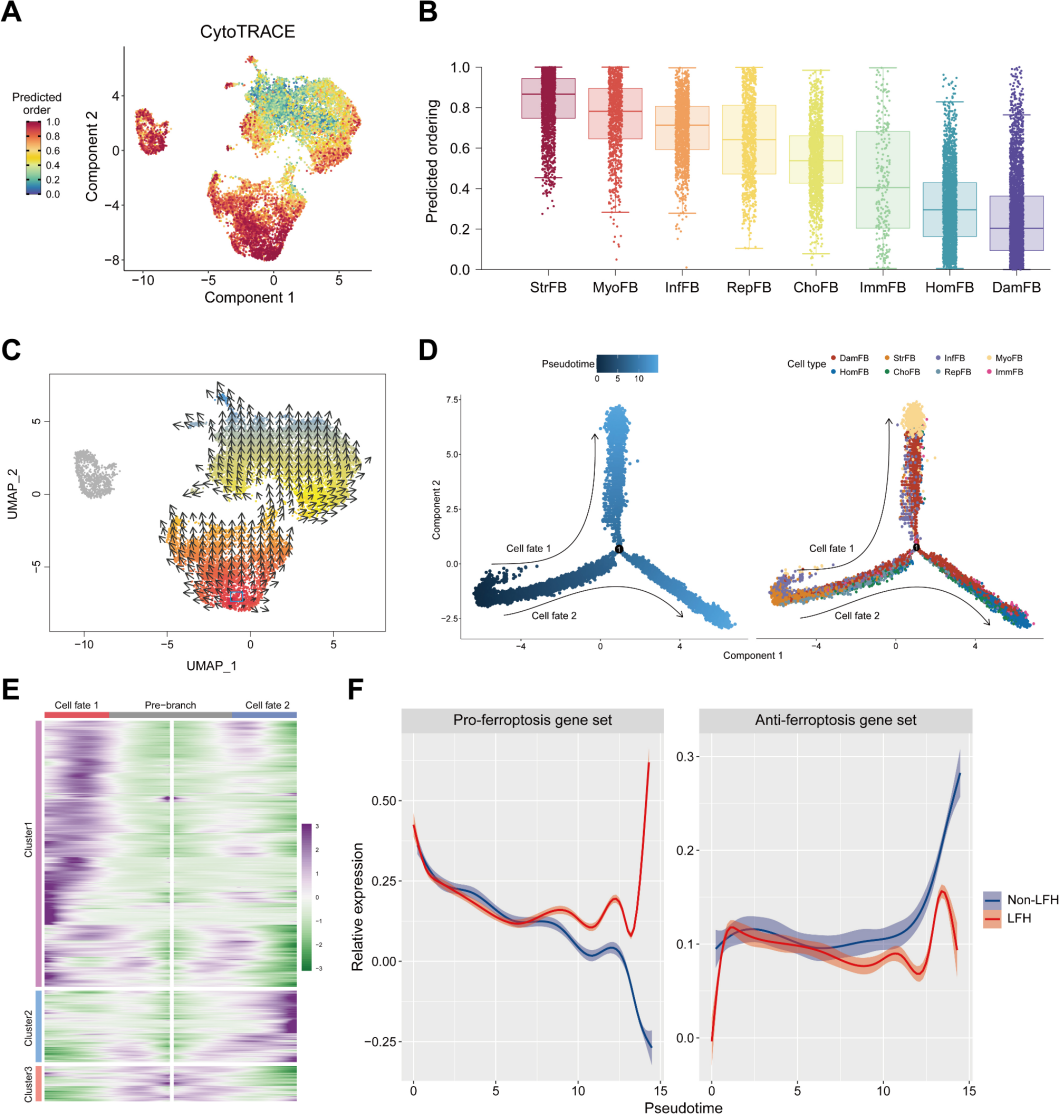
Pseudotime analysis reveals the cell development trajectories of fibroblast subpopulations in LFH. **(A)** UMAP plot showing the distribution of CytoTRACE scores among fibroblast subpopulations. Dark blue represents lower scores (highly differentiated), while dark red indicates higher scores (less differentiated). **(B)** Box plot presenting the distribution of CytoTRACE scores across different fibroblast subsets. The level of differentiation is represented by the color gradient, from blue (highly differentiated) to red (less differentiated). **(C)** Single-cell RNA dynamics plot for fibroblast subpopulations showing cells within the blue rectangle as Starting Cells, with arrows indicating predicted developmental trajectories. Pseudotime order is illustrated by the color gradient from red (early pseudotime) to blue (late pseudotime). **(D)** Developmental pseudo-time (left) and the distribution of different cell types (right) in LF fibroblast subtypes were generated using Monocle2. Darker blue represents earlier pseudo-time. Each fibroblast subtype is labeled with a distinct color. Arrows indicating the differentiation directions of two cell fates: cell fate 1 and cell fate 2, where cell fate 1 represents MyoFB, and cell fate 2 represents ChoFB. **(E)** Heatmap showing expression profiles of different cell fates along pseudotime. DEGs (qval < 1e-5) are hierarchically clustered into three subclusters. **(F)** Shaded line plot showing the expression scores of pro-ferroptosis and anti-ferroptosis gene sets over pseudotime in non-LFH (blue) and LFH (red) samples

After the clustering and functional analyses, we aimed to describe their developmental trajectories and cell fate transitions through pseudotime analysis. First, we utilized the CytoTRACE algorithm to identify the origins of fibroblast subpopulations (Fig. 4A). This algorithm can predict relative cell states and differentiation directions by reconstructing cell trajectories based on gene counts and expression. The CytoTRACE scores for each fibroblast cluster (Fig. 4B) revealed that MyoFB and StrFB have the highest stemness and the lowest differentiation levels [23]. However, DamFB exhibited the highest differentiation level, suggesting it may represent the final state of fibroblast subpopulation. Previous studies have indicated that myofibroblasts emerge from fibroblasts during the LF fibrosis process and are characterized by stemness-associated genes such as *ACTA2* and *MYLK*. Based on these results and the single-cell RNA dynamics map (Fig. 4C) obtained using the VECTOR method [24], we proposed that StrFB represents the functional state of the normal LF tissue and serves as the origin for fibroblast subpopulations.

Subsequently, we constructed the developmental trajectories of all fibroblast subpopulations in LF tissue through Monocle2 [25]. The pseudotime analysis results showed that the trajectory started with StrFB and then diverged into two main branches (Fig. 4D). The heatmap displays the expression profiles of different cell fates at each branch (Fig. 4E). Notably, MyoFB and ChoFB are positioned at opposite divergent ends, while DamFB is distributed at both ends of the trajectory (Fig. 4D). This indicated that during the LFH process, fibroblasts follow two primary differentiation pathways: one leads to the activation of myofibroblasts and the other to the differentiation into chondrocyte-like cells. And the damaged frailty-specific fibroblasts existed at both ends of these differentiation pathways. To reveal the molecular dynamics of ferroptosis in LFH fibrosis, we explored the expression patterns of pro-ferroptosis and anti-ferroptosis gene sets across different groups over pseudotime (Fig. 4F). Notably, in the LFH group, pro-ferroptosis gene expression continuously increased with pseudotime, while anti-ferroptosis genes showed progressively decreasing expression scores. In contrast, the non-LFH group exhibited a decrease in pro-ferroptosis gene expression over pseudotime, whereas an increase in anti-ferroptosis gene expression. These findings suggest that during LF degeneration, ferroptosis-promoting genes aggravate disease progression, while ferroptosis-resisting genes play a protective role in delaying the degenerative process.

### Increased ferroptosis levels correlate with elevated fibrosis in LFH in both human specimens and mouse models

To further investigate the involvement of ferroptosis in LFH fibrosis, we analyzed the expression of some ferroptosis-associated key genes through scRNA-seq. *GPX4* is a crucial gene that protects cells from ferroptosis. Its knockdown or inhibition will impair the cell’s ability to scavenge phospholipid peroxides, leading to the susceptibility to ferroptosis in various cell types [43]. The *FTL* gene encodes the ferritin light chain, a critical component of ferritin that plays an important role in iron metabolism by storing and sequestering excess free intracellular iron, thus inhibiting the onset and progression of ferroptosis [44, 45]. *ACSL4* encodes an enzyme that provides polyunsaturated fatty acid-containing phospholipids as substrates for lipid peroxidation, thereby promoting ferroptosis [46]. To characterize the association between ferroptosis and fibrosis in LFH, marker genes of fibrosis [47], including *COL1A2*, *FN1*, and *ACTA2*, were also evaluated in the scRNA-seq analysis. Elevation of *COL1A2*, which encodes Collagen I (COL1), is a significant marker of fibrosis in multiple organs, including LFH. *FN1* encodes Fibronectin, a pro-regenerative gene and an important component of the ECM transformation and activation in LFH [47].

**Fig. 5:**
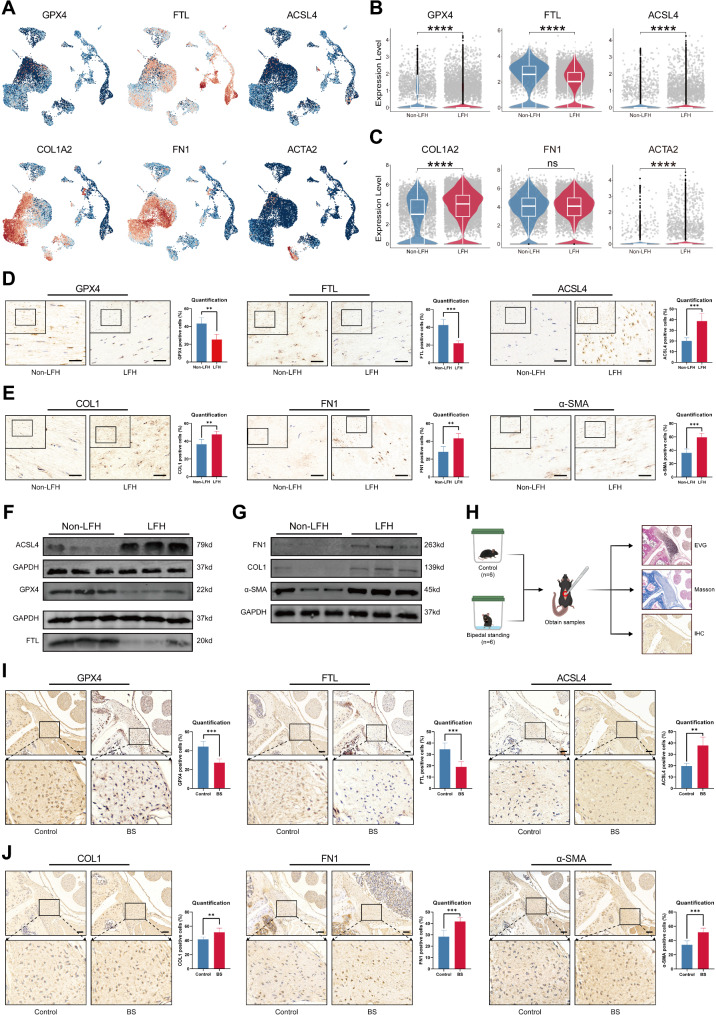
Ferroptosis levels increased with fibrosis in LFH in both human specimens and mouse models. **(A)** UMAP plots showing the expression levels of ferroptosis markers (*GPX4*, *FTL*, *ACSL4*) and fibrosis markers (*COL1A2*, *FN1*, *ACTA2*). Red indicates the high expression, and blue indicates the low expression. (**B**, **C**) Violin plots illustrating the expressions of *GPX4*,* FTL*, *ACSL4*, *COL1A2*, *FN1*, and *ACTA2* in fibroblasts from non-LFH and LFH samples. ‘ns’ represent *p* > 0.05, ****p < 0.0001. **(D)** Representative IHC staining for GPX4, FTL, and ACSL4 in non-LFH and LFH samples (*n* = 5). Scale bar, 50 µm. Data quantification results are shown on the right, expressed as mean ± SD. **(E)** Representative IHC staining for COL1, FN1, and α-SMA in non-LFH and LFH samples (*n* = 5). Scale bar, 50 µm. Data quantization results are shown on the right. **(F)** Western blot analysis of GPX4, FTL, and ACSL4 protein expression in non-LFH and LFH samples (*n* = 3). **(G)** Western blot analysis of COL1, FN1, and α-SMA protein expression in non-LFH and LFH samples (*n* = 3). **(H)** Schematic illustration of the BS mouse model used to induce LFH. **(I)** Representative IHC staining for GPX4, FTL, and ACSL4 in control and BS mouse samples (*n* = 6). **(J)** Representative IHC staining for COL1, FN1, and α-SMA in control and BS mouse samples (*n* = 6). Scale bar, 50 µm. Data quantification results are shown on the right and displayed as mean ± SD. ***p < 0.001, ****p < 0.0001

The UMAP plots illustrate the expression levels of ferroptosis markers (*GPX4*, *FTL*, *ACSL4*) and fibrosis markers (*COL1A2*, *FN1*, *ACTA2*) (Fig. 5A). We found that the expression levels of *GPX4* and *FTL*, which inhibit ferroptosis, were significantly reduced in fibroblasts from the LFH group. *ACSL4*, however, a promoter of ferroptosis, showed notably higher expression in these fibroblasts (Fig. 5B). Additionally, the results of the scRNA-seq showed that the expression levels of fibrosis markers *COL1A2*, *FN1*, and *ACTA2* were markedly increased in fibroblasts from the LFH group (Fig. 5C). To further evaluate the critical role of ferroptosis in LFH, we validated the scRNA-seq results using clinical specimens. Immunohistochemistry (IHC) analysis (Fig. 5D-E) and Western blot results (Fig. 5F-G) demonstrated that in hypertrophied human LF tissue, the fibrosis markers COL1, FN1, and α-SMA were significantly elevated. Moreover, the expression levels of the pro-ferroptosis molecule ACSL4 were significantly increased in the LFH group, while the levels of GPX4 and FTL, known to inhibit ferroptosis, were notably decreased.

Additionally, we utilized a BS model as a mouse model of LFH for further validation (Fig. 5H) [31]. Compared to the control group, the results of EVG and Masson staining showed that mice in the BS group exhibited a remarkable decrease in the proportion of elastic fibers and a significant increase in the proportion of collagen fibers after 12 weeks of modeling (Additional file 7: Fig. S5). These findings indicated that the LFH degeneration model was effectively established in this group of mice. In the IHC staining analysis of mice (Fig. 5I-J), the findings were consistent with those observed in human clinical specimens. The percentage of positive cells for the fibrosis markers COL1, FN1, and α-SMA, as well as the pro-ferroptosis molecule ACSL4, was significantly upregulated in the BS group compared to the control group. In addition, the percentage of positive cells for GPX4 and FTL, which inhibit ferroptosis, was notably decreased in the BS group. These results indicate a significant increase in ferroptosis in hypertrophic LF tissue undergoing fibrosis, compared to normal LF tissue. Based on these results, we hypothesized that ferroptosis may be involved in the fibrotic process of LFH and play a crucial role in promoting disease progression.

### Integrated analysis of transcriptomics and BayesPrism deconvolution uncovers the close association between macrophages and fibroblasts

**Fig. 6:**
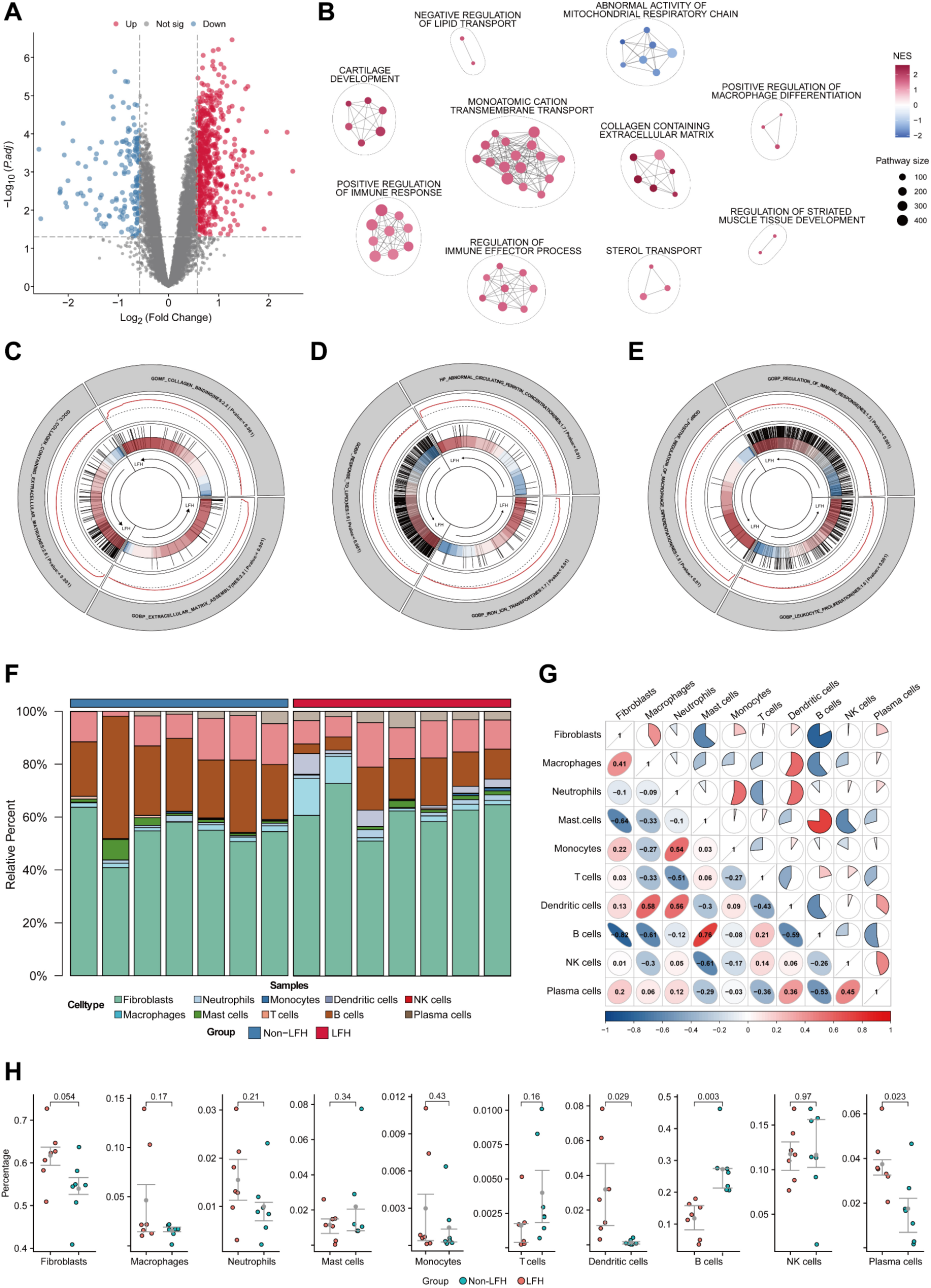
Integrated analysis of transcriptomics and BayesPrism deconvolution uncovers the close association between macrophages and fibroblasts. **(A)** Volcano plot showing the DEGs between the LFH group and the non-LFH group in the merged bulk RNA-seq data (|FC| > 1.5, *p* value < 0.05). Red dots indicate up-regulated LFH genes, blue dots indicate down-regulated LFH genes, and gray dots indicate genes with no significant differences. **(B)** Network Diagrams indicating network clustering based on GSEA gene set enrichment. The network was composed of enriched pathways with *p* < 0.05, with the circle sizes indicating the number of genes within each pathway. The color of the diagram reflects the NES, with red indicating positive and blue indicating negative. (**C**, **D**, **E**) GSEA circular plots showing the enrichment results of gene sets related to ECM and collagen formation, gene sets related to iron metabolism and lipid metabolism, and gene sets related to immunity and macrophages. **(F)** Bar plot indicating the cell composition proportions of each sample in the bulk RNA-seq data after BayesPrism deconvolution. **(G)** Heatmap showing the Spearman correlation analysis results between different cell types. Red indicates a positive correlation, while blue indicates a negative correlation. **(H)** Bar graphs illustrating the differences in cell proportions between the non-LFH group and the LFH group in the bulk RNA-seq data after deconvolution analysis. Red dots represent LFH group samples, while blue dots represent non-LFH group samples

To increase the sample size of sequencing data and enhance the feasibility of single-cell analysis, we further utilized LF samples from 6 donors (3 LF samples from the non-LFH group and 3 LF samples from the LFH group) for RNA-seq. The clinical information of donors for RNA-seq is included in Additional file 3: Table S3. Additionally, we retrieved the microarray dataset GSE113212 from the GEO, which includes 4 LFH samples from elderly individuals and 4 non-hypertrophic samples from younger individuals. After combining these two datasets, the PCA results indicated that batch effects were well corrected, showing good differentiation between the two sample groups (Additional file 7: Fig. S6). Differential expression analysis of the merged bulkRNA-seq data identified a total of 711 DEGs, including 544 upregulated and 167 downregulated genes (Fig. 6A). To identify the biological processes significantly enriched during LFH pathology progression in the bulk RNA data, we conducted a GSEA on all genes. The GSEA network diagram displayed the overall pathway enrichment of gene sets (Fig. 6B), highlighting pathways such as “monoatomic cation transmembrane transport”, “collagen-containing extracellular matrix” and “positive regulation of macrophage differentiation”. The circular diagram provided more detailed GSEA pathway enrichment results (GSEA results are provided in Additional file 6: Table S6). We observed that LFH group samples were enriched in terms closely related to fibrosis, including “collagen binding”, “collagen-containing extracellular matrix”, and “extracellular matrix assembly” (Fig. 6C). Furthermore, biological processes related to iron and lipid metabolism, such as “iron ion transport”, “abnormal circulating ferritin concentration” and “response to lipid” were also enriched in the LFH group (Fig. 6D). Notably, pathways related to immunity and macrophages, such as “regulation of immune response”, “positive regulation of macrophage differentiation” and “leukocyte cell-cell adhesion” were also upregulated in the LFH group (Fig. 6E).

We then performed a deconvolution analysis with the standard workflow of BayesPrism [26]. BayesPrism is a novel deconvolution method that utilizes scRNA-seq data as prior information to perform cell type and gene expression deconvolution in merged bulk RNA-seq data, allowing us to infer the composition and expression profiles of fibroblasts and immune cells in LF tissue. Subsequently, correlation analysis of cell types and states from scRNA-seq showed dissimilar clusters (Additional file 7: Fig. S7A), limiting the use of combined analysis. Additionally, we identified outlier genes, including ribosomal, mitochondrial, sex chromosome, and low-transcription genes, and excluded them from further analysis (Additional file 7: Fig. S7B). Focusing on “protein-coding genes”, we constructed a Prism object and derived cell composition from BayesPrism (Fig. 6F). As expected, many cell types showed similar proportional changes between bulk RNA-seq and scRNA-seq data. Compared to the non-LFH group, the LFH group showed increased proportions of fibroblasts and macrophages, and statistically significant changes were also observed in the proportions of B cells, plasma cells, and dendritic cells (Fig. 6H). Although the changes in the proportions of fibroblasts and macrophages were not statistically significant, we still believe that fibroblasts and macrophages play crucial roles in the biological processes of LFH because of the heterogeneity of cell types between samples and biological variability among patients. Furthermore, Spearman correlation analysis revealed that fibroblasts exhibited the highest positive correlation with macrophages (Fig. 6G), suggesting that macrophages may exert effects in the LFH degeneration process through interactions with fibroblasts, which may be closely related to the pathogenesis of fibrosis in LFH. In summary, our findings from bulk RNA-seq are consistent with those from scRNA-seq enrichment analysis. Based on the changes in cell type proportions observed in BayesPrism deconvolution and the results of Spearman correlation analysis, we speculated that a close association may exist between macrophages and fibroblasts, potentially playing a crucial role in the disease progression of LFH.

### Mononuclear phagocytic system lineage analysis reveals the potential function of SPP1+ Mac enriched in LFH

**Fig. 7:**
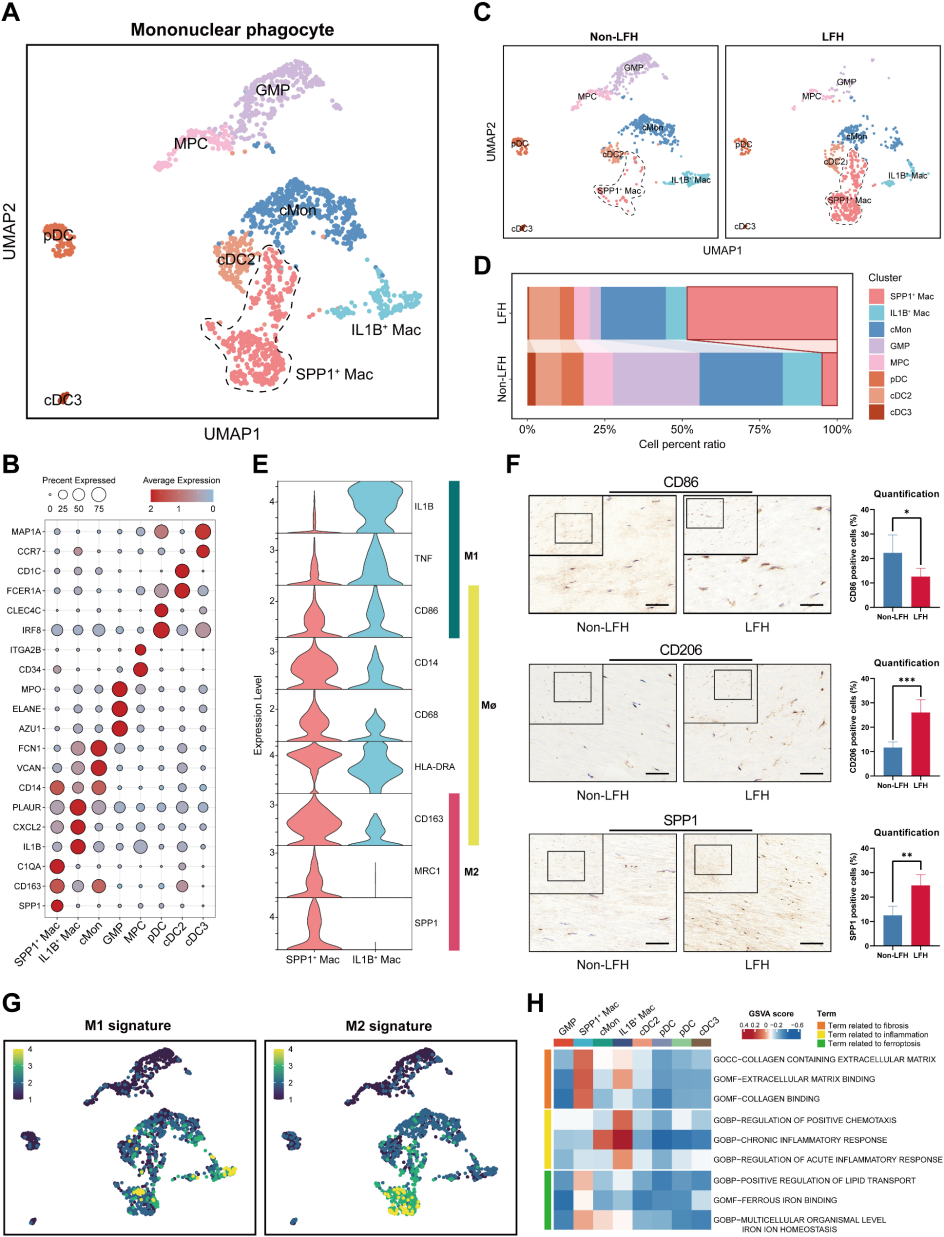
Mononuclear phagocytic system lineage analysis reveals the potential function of SPP1^+^ Mac enriched in LFH. **(A)** UMAP of MPs in human LF tissue, dyed according to the cell types. SPP1^+^ Mac are circled by the black dotted line. **(B)** Dot plot displaying marker gene expression in different mononuclear phagocyte subpopulations, with color indicating the scaled mean expression of genes. **(C)** UMAP of MPs from the non-LFH group and the LFH group, dyed according to the cell types. **(D)** Bar graph showing the proportion of each mononuclear phagocyte subpopulation in the non-LFH group and the LFH group. **(E)** Stacked violin plot showing the expression of common markers in SPP1^+^ Mac and IL1B^+^ Mac, categorized into Mø, M1, and M2 types. **(F)** Representative IHC images showing the expression of CD86, CD206, and SPP1 in non-LFH and LFH samples. Scale bar, 50 μm. Data quantification results are presented on the right, as mean ± SD, with *p < 0.05, **p < 0.01, and ***p < 0.001. **(G)** UMAP plots showing M1 (left) and M2 (right) scoring results for each mononuclear phagocyte cluster, with yellow representing high scores and purple representing low scores. **(H)** Heatmap revealing GSVA scoring results for each mononuclear phagocyte cluster, with red indicating high scores and blue indicating low scores

Considering that the initial clustering lacked a detailed grouping of macrophages, we extracted MPs (including monocytes, macrophages, dendritic cells, and GMPs) for a second round of clustering (Fig. 7A). Specifically, we annotated and identified 8 precise subgroups based on myeloid immune cell markers (Fig. 7B): cMon (classical monocytes, highly expressing *CD14* and *VCAN*), SPP1^+^ Mac (highly expressing *C1QA* and *SPP1*), IL1B^+^ Mac (IL1B^+^ macrophages, highly expressing *IL1B* and *CXCL2*), GMP (granulocyte monocyte progenitor, highly expressing *AZU1* and *ELANE*), MPC (megakaryocyte progenitor cells, highly expressing *CD34* and *ITGA2B*), pDC (Plasmacytoid dendritic cells, highly expressing *IRF8*, *LILRA4*, and *CLEC4C*), cDC2 (Conventional dendritic cells type 2, highly expressing *FCER1A*, *CD1C*, and *CD1E*), and cDC3 (Conventional dendritic cells type 3, highly expressing *CCR7* and *MAP1A*). Interestingly, SPP1^+^ Mac, a novel subpopulation not previously reported in LFH studies, were present in small numbers in the non-LFH group. However, in the LFH group, both the number and proportion of SPP1^+^ Mac increased significantly (Fig. 7C-D). These findings suggest a shift in macrophage polarization in hypertrophic LF tissue, with SPP1^+^ Mac potentially playing a crucial role in the progression of LFH.

Notably, we found that these two newly classified macrophage subtypes, SPP1^+^ Mac and IL1B^+^ Mac, exhibit distinctly different molecular characteristics and biological functions. Then we analyzed their expressions of common markers for macrophages (Mø), classically activated macrophages (M1), and alternatively activated macrophages (M2) (Fig. 7E). SPP1^+^ Mac showed high expression of M2 markers *CD163* and *MRC1*(*CD206*), whereas IL1B^+^ Mac exhibited higher expression of M1 markers *TNF* and *CD86*. Subsequently, IHC results showed higher expression levels of CD86, CD206, and SPP1 in the LFH group (Fig. 7F), indicating that both M1 and M2 macrophage populations proliferate during the LFH fibrosis process, with a more significant increase in SPP1^+^ Mac. Consistent with our findings, previous studies have reported that M1 promote inflammation in fibrotic diseases, whereas M2 primarily function in tissue repair and pro-fibrotic processes [48]. Interestingly, the M1 and M2 scores of all MPs showed (Fig. 7G) that SPP1^+^ Mac had a higher M2 score, while IL1B^+^ Mac had a higher M1 score. GSVA results also supported this finding (Fig. 7H). Fibrosis-related GO terms such as “collagen containing extracellular matrix”, “extracellular matrix binding” and “collagen binding” were enriched in SPP1^+^ Mac. However, IL1B^+^ Mac were more enriched in inflammation-related biological processes like “regulation of positive chemotaxis”, “chronic inflammatory response” and “regulation of acute inflammatory response”. Our analysis suggested that SPP1^+^ Mac may have a potential pro-fibrotic function, while IL1B^+^ Mac may be more involved in inflammation regulation. Interestingly, SPP1^+^ Mac also exhibited stronger activity in pathways related to iron and lipid metabolism, such as “positive regulation of lipid transport”, “ferrous iron binding” and “multicellular organismal level iron ion homeostasis”. This indicated that SPP1^+^ Mac may play a critical role in regulating ferroptosis in fibroblasts during LFH pathology progression.

In conclusion, we provided a more detailed depiction of the mononuclear phagocyte atlas in LFH and explored the functions of macrophages. More importantly, we identified SPP1^+^ Mac as being closely associated with LFH disease progression. However, the mechanisms by which SPP1^+^ Mac promote LFH fibrosis and regulate ferroptosis still require further investigation.

### Deciphering the complex interactions among multiple cell lineages in the fibrotic microenvironment of LFH

**Fig. 8:**
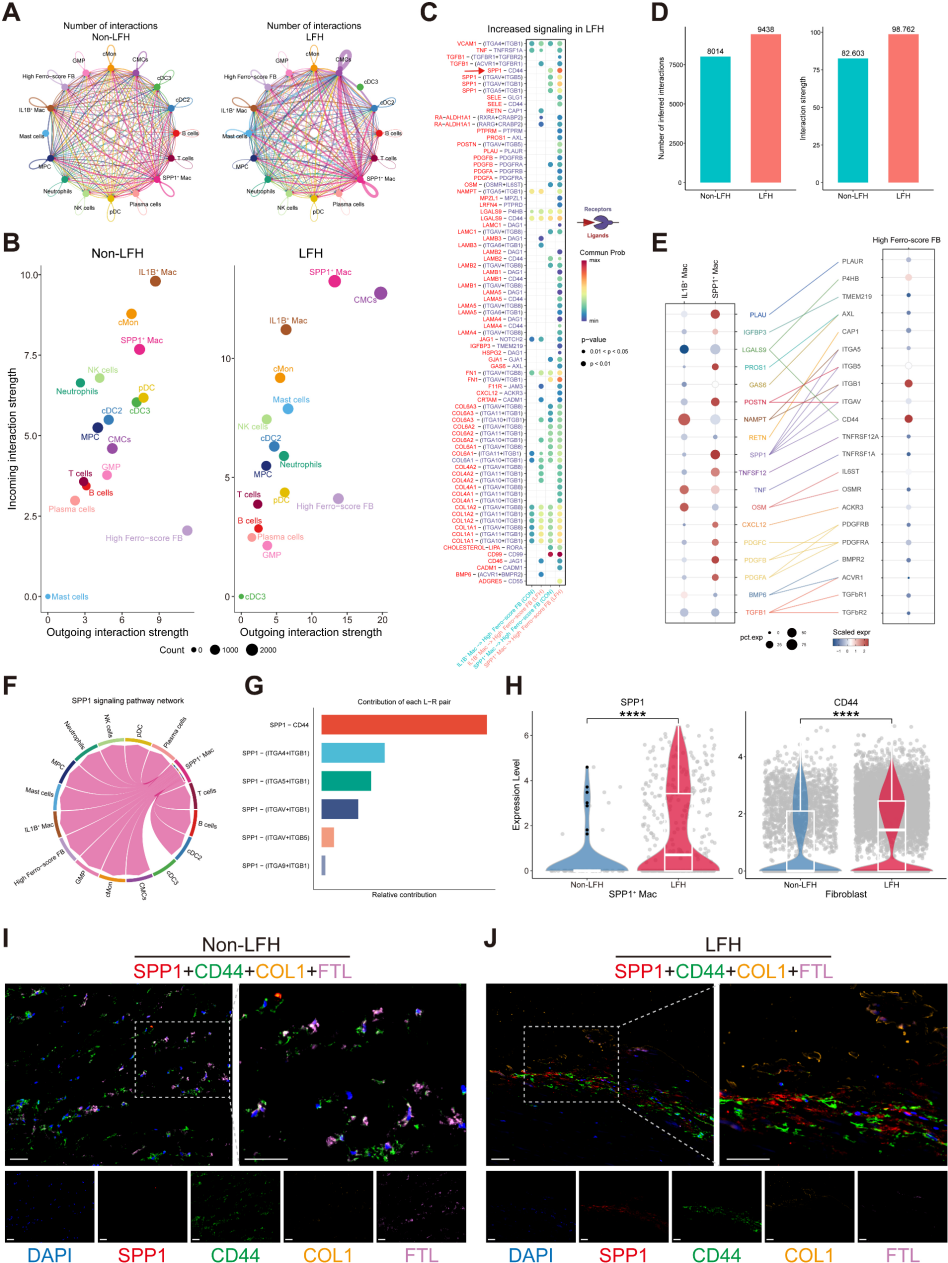
Deciphering the complex interactions among multiple cell lineages in the fibrotic microenvironment of LFH. **(A)** Circos plots showing potential interactions between High Ferro-score FB and 15 immune cell clusters in the non-LFH (left) and LFH groups (right). Edge width indicates the number of significant L-R pairs between cell types. **(B)** Interaction strengths for incoming and outgoing signaling events among all clusters in non-LFH and LFH groups. The horizontal axis represents outgoing interaction strength, while the vertical axis represents incoming interaction strength. **(C)** Bubble plot illustrating communication probabilities of L-R interactions between SPP1^+^ Mac or IL1B^+^ Mac subclusters (sending signals) and High Ferro-score FB subcluster (receiving signals) in the up-regulated signaling pathways of the LFH group.Red characters represent ligands, and purple characters represent receptors. Bubble color and size represent calculated communication probabilities and *p*-values, respectively. **(D)** Comparison of the number and strength of inferred cellular interactions in the non-LFH and LFH groups. **(E)** Bubble connectivity plot displaying upregulated receptor-ligand pairs from signaling pathways in the LFH group, and their expression levels in corresponding cell clusters. The color of the bubble reflects the communication probability, and the bubble size indicates the percentage expression of L-R pairs. **(F)** Circle plot of inferred SPP1 signaling networks among SPP1^+^ Mac and other cell clusters. **(G)** Bar graph showing the distribution of L-R pairs between SPP1^+^ Mac and High Ferro-score FB. **(H)** Violin plots showing the expression difference of ligand SPP1 between SPP1^+^ Mac in non-LFH and LFH groups, and of receptor CD44 expression in High Ferro-score FB between non-LFH and LFH groups. ****p < 0.0001. (**I**-**J**) Representative multiplex immunofluorescent images of LF tissue from non-LFH and LFH groups showing the expression of SPP1 (red), CD44 (green), COL1 (orange), and FTL (purple). Nuclei are labeled with DAPI (blue). Scale bar, left, 100 μm; bottom, 100 μm; right, 20 μm

Immune cells are key regulators of LFH. Therefore, understanding their interactions with fibroblasts within the fibrotic microenvironment of LFH is crucial for unraveling the pathogenesis of the disease. We extracted High Ferro-score FB and all immune cells from the previous ferroptosis score for cell-cell communication analysis. Potential interactions between different cell types revealed significant differences in cellular communication networks between the LFH and non-LFH groups (Fig. 8A). The LFH group exhibited a greater number of interactions and stronger interaction strengths, indicating more complex and robust interactions in hypertrophied LF tissue (Fig. 8D). Comparing the interaction strengths of signaling pathways for each cell cluster in both groups, we found that High Ferro-score FB, IL-1B^+^ Mac, SPP1^+^ Mac all exhibited strong interactions within the local LF microenvironment. In the non-LFH group, IL-1B^+^ Mac demonstrated stronger incoming and outgoing interaction strength compared to the SPP1^+^ Mac. In contrast, in the LFH group, both the incoming and outgoing interaction strengths of SPP1^+^ Mac surpassed those of IL-1B^+^ Mac. These suggested that SPP1^+^ Mac gradually replaces IL1B^+^ Mac as the primary intercellular signals during LFH fibrosis, exerting a more pronounced regulating influence (Fig. 8B). Additionally, we identified all signaling pathways upregulated during LFH progression (Fig. 8C), using SPP1^+^ Mac and IL1B^+^ Mac as signal senders and High Ferro-score FB as target cells receiving signals. Notably, we observed significant increases in the communication probability of the SPP1 signaling pathway, as well as fibrosis-related pathways such as FN1, PDGF, TGFB1, and collagen-related COL1A2 in the LFH group compared with the non-LFH group (Fig. 8C). This suggested that the increased fibrosis level observed in hypertrophied LF tissue compared to normal tissue may be driven by SPP1^+^ Mac regulation. Further detailed analysis of upregulated signaling pathways mediated by secreted factors is demonstrated in the LFH group (Fig. 8E). We found that *NAMPT*, associated with nicotinamide adenine dinucleotide (NAD^+^) biosynthesis [49], was most highly expressed as a ligand in IL1B^+^ Mac, while its receptor ITGB1 was most highly expressed in High Ferro-score FB target cells. Additionally, among the ligands of SPP1^+^ Mac, SPP1, which is associated with reparative macrophage polarization [50], was the most strongly expressed. And its receptor CD44 was the most highly expressed in target cells (Fig. 8E). These findings indicate that the NAMPT signaling pathway is primarily regulated by IL1B^+^ Mac, whereas the SPP1 signaling pathway may serve as the main cellular communication mode between SPP1^+^ Mac and High Ferro-score FB.

Then we analyzed the SPP1 signaling pathway and found that it is exclusively sent by SPP1^+^ Mac, interacting with multiple cell types (Fig. 8F). Analysis of ligand-receptor (L-R) pairs between SPP1^+^ Mac and High Ferro-score FB revealed that the SPP1-CD44 pair was the most prominent contributor to the SPP1 signaling pathway (Fig. 8G). In hypertrophic LF samples, the expression of the receptor CD44 was significantly upregulated in High Ferro-score FB, while the ligand SPP1 was similarly more highly expressed in SPP1^+^ Mac (Fig. 8H). Multiplex immunofluorescent analysis further confirmed that SPP1 and CD44 expression was markedly elevated in hypertrophied LF samples (Fig. 8I-J), consistent with our scRNA-seq results. Interestingly, in LFH samples, we observed substantial COL1 deposition in regions where SPP1 and CD44 co-localized, along with reduced expression of the iron death resistance gene *FTL* in fibroblasts. In contrast, normal LF samples exhibited much less SPP1-CD44 binding, with a corresponding decrease in COL1 deposition and a significant increase in FTL expression. These findings suggested that SPP1^+^ Mac may regulate ferroptosis in fibroblasts through the SPP1-CD44 signaling axis, thereby altering the fibrotic microenvironment in LF tissue and promoting disease progression in LFH.

In summary, our findings present a cellular crosstalk network involving multiple signaling pathways between High Ferro-score FB and various immune cells, revealing potential pathways contributing to LF fibrosis. Notably, SPP1^+^ Mac appear to play a crucial role in driving LFH fibrosis via the SPP1-CD44 axis of the SPP1 signaling pathway, likely by potentially regulating ferroptosis in fibroblasts.

## Discussion

LFH can lead to numbness and pain in the lower limbs of patients and, in severe cases, result in incontinence and paralysis, posing a substantial burden on the healthcare system and society [51]. However, the cellular and molecular mechanisms underlying LFH remain incompletely understood. Concurrently, because of the limitations of previous techniques and analyses, the cell composition and gene expression profiles of LFH at single-cell resolution have not been fully elucidated. Therefore, establishing a detailed single-cell landscape of human LF tissue is essential for understanding the tissue characteristics of LF and the potential mechanisms of LFH.

In this study, we have made some novel and important findings. We performed multi-omics analyses, including scRNA-seq and bulk RNA-seq on human LF tissue. These analyses revealed the genetic characteristics and distribution of various cell populations involved in LF fibrosis. Previous studies have applied scRNA-seq to investigate connective tissue such as ligaments and tendons, including the posterior longitudinal ligament [52], anterior cruciate ligament [37], and various tendons [53], and have determined some molecular markers of ligament issues. Leveraging these markers, we characterized 19 distinct cell subpopulations in LF tissue, including fibroblasts, myofibroblasts, and various immune cells such as macrophages. Furthermore, we employed deconvolution methods to infer cell types from bulk transcriptome samples due to the sample insufficiency because of the high costs for scRNA-seq and the difficulty in fully dissociating clinical specimens into single-cell suspensions. This multi-omics-based analysis, which integrated bulk RNA-seq with scRNA-seq, allowed for more precise inference of cell type abundance, providing a foundation for identifying disease-associated cell types in LF tissue.

Ferroptosis, first proposed in 2012 [54], is defined as a regulated cell death driven by iron-mediated excessive lipid peroxidation [55]. In recent years, extensive research has been conducted on the critical role of ferroptosis in various organ fibrosis [20]. For instance, in one study on pulmonary fibrosis, TGF-β1 was found to promote the transcription of *TFRC* in lung fibroblasts, leading to iron overload. And ferroptosis inhibitors combined with iron chelators and specific *TFRC* knockout have been shown to alleviate pulmonary fibrosis in mice [56]. In another study on myocardial fibrosis, researchers identified MLK3-induced ferroptosis as a crucial process in pressure overload-induced myocardial fibrosis [57]. Additionally, investigations into renal fibrosis have indicated that downregulating *ACSL4* can mitigate TGF-β1-induced ferroptosis and the fibrotic phenotype [58]. Nevertheless, the role of ferroptosis in LFH remains not fully understood. In this study, through scRNA-seq and transcriptome functional analysis, together with MR analysis, we found that ferroptosis-related pathways were significantly upregulated in LFH, along with fibrosis-related pathways. This suggests that ferroptosis may act as a risk factor for fibrosis in LFH. MR facilitates us to elaborate the potential mechanism of LFH clinically by inferring causal associations between exposure factors and outcomes utilizing SNPs as instrumental variables [59]. These methods, integrating population-based epidemiological analyses with single-cell biology research strategies, enhance the reliability of single-cell analysis and facilitate the transition of LFH research from a molecular biology perspective to an epidemiological one.

By integrating functional clustering analysis of LF fibroblasts with ferroptosis score, we established a subpopulation distribution map of High Ferro-score FB and analyzed metabolic reprogramming related to ferroptosis in fibroblasts. These metabolic changes involving iron metabolism, lipid metabolism, and redox homeostasis weakened the ferroptosis resistance across multiple subpopulations of fibroblasts, indicating that ferroptosis tendency serves as a background for the pro-fibrotic function of fibroblasts. To further explore the role of ferroptosis in the progression of LFH, we performed pseudotime analysis on LF fibroblasts based on scRNA-seq to identify the differentiation direction of each subpopulation, and depicted the evolution of ferroptosis-related gene expression with pseudo temporal changes. Notably, the expression of pro-ferroptosis genes increased with the evolution of fibroblast functional differentiation during LFH, while the expression of anti-ferroptosis genes decreased. To further validate the role of ferroptosis in LFH, we initially identified ferroptosis in fibroblasts during the progression of LFH using TEM. Complementary findings from EVG and Masson staining further revealed that LF specimens from the LFH group exhibited not only increased fibrosis but also more characteristics of ferroptosis under TEM. Ferroptosis is primarily indicated by mitochondrial shrinkage, with denser membranes and a reduction of cristae [60]. These characteristic changes are readily observable under TEM, establishing it as a direct and critical method for identifying ferroptosis. Specifically, the IHC results showed that in degenerated hypertrophic LF tissue, the expression level of the pro-ferroptosis gene *ACSL4* significantly increased because of advancing fibrosis levels, while the expression levels of anti-ferroptosis genes *GPX4* and *FTL* notably decreased as fibrosis progressed. These findings indicate that ferroptosis is a pivotal participant in the progression of LFH and may play a significant role in the promotion of fibrosis. Importantly, we also utilized the previously reported BS mouse model from our research group for in vivo experimental validation [21, 31]. In this model, mice spontaneously lifted their forelimbs due to hydrophobicity in a water-filled apparatus, thereby stimulating the mechanical stress on the lumbar spine during prolonged human standing. The BS model, being non-invasive, aligned more closely with the physiological degeneration process of the human lumbar spine compared to other models. Thus, the above exploration of ferroptosis via single-cell analysis, complemented by experimental verification, is necessary to enhance our understanding of the fibrotic mechanisms in LFH and identify ferroptosis as a key characteristic during LFH progression. As we delve deeper into the mechanisms of ferroptosis in LFH, inhibiting ferroptosis may emerge as a promising therapeutic strategy to decelerate LFH degeneration and fibrosis.

In this study, we found a significant increase in the proportions of myofibroblasts and macrophages in LFH samples. Myofibroblasts are the main effector cells of LFH fibrosis, and the activation of fibroblasts into myofibroblasts is a key cellular event driving the progression of LFH [9, 34]. In renal interstitial fibrosis, ferroptosis of renal tubular epithelial cells has been demonstrated to activate the transformation of fibroblasts into myofibroblasts through paracrine profibrotic factors [61]. In idiopathic pulmonary fibrosis, GPX4 levels in pulmonary fibroblasts are reduced, and *GPX4* knockdown significantly enhances TGF-β-induced SMAD2/3 activation, thereby promoting myofibroblast differentiation in lung fibroblasts [62]. These findings suggest that the upregulation of ferroptosis in fibroblasts may enhance their tendency to transform into myofibroblasts, thereby exacerbating LFH fibrosis. Notably, BayesPrism and Spearman correlation analyses identified macrophages as the cell type most strongly correlated with fibroblasts, highlighting a close interaction between these populations in LFH. Therefore, we further explored the potential role of macrophages in regulating LFH fibrosis by promoting ferroptosis in fibroblasts. As crucial components in inflammation and injury repair processes, macrophages are key regulators in the initiation and progression of fibrosis [14, 63]. Macrophage phenotypes exhibit distinct characteristics at various tissue repair stages, and functional imbalances among these phenotypes can drive tissue repair toward an aberrant fibrotic process [64, 65]. In order to reveal the characteristics and evolution of macrophage phenotype during the progression of LFH in more detail, following the reclustering of MPs, we identified two distinct macrophage subtypes exhibiting divergent functions and specific markers in LFH. Previous studies have shown that early macrophages recruited in response to tissue injury primarily exhibit a pro-inflammatory phenotype [64]. They can aggravate local inflammation through phagocytosis of cells and tissue debris, the release of damaging substances, and the secretion of inflammatory mediators [65, 66]. After the inflammation resolution, the pro-repair or the pro-fibrotic phenotype becomes the dominant characteristic of the macrophage population [48], which is consistent with our findings. Through single-cell gene set scoring, GSVA analyses, and IHC, we found that IL1B^+^ Mac present a pro-inflammatory M1 phenotype, while SPP1^+^ Mac were significantly increased in hypertrophic LF samples and exhibited a pro-fibrotic M2 phenotype.

This type of macrophages, capable of both regulating ferroptosis and driving fibrosis, is SPP1^+^ Mac, a novel subtype that has not been previously reported in LFH. Recently, increasing attention has been placed on SPP1^+^ Mac. As a hallmark of myofibroblast formation, osteopontin (OPN), encoded by SPP1, is closely related to tissue fibrosis such as inflammatory cell chemotaxis, fibroblast activation and ECM remodeling [37, 67]. In addition, the role of SPP1^+^ Mac has been identified in studies of fibrosis across various organs, such as the lung [68], heart [69], and tendon [53], suggesting that they may represent a cross-organ and cross-species pro-fibrotic macrophages subtype. These studies also align with our findings. The analyses of GSVA enrichment and IHC demonstrated that SPP1^+^ Mac also contribute to fibrosis progression in LFH. Recent studies have found that macrophages contribute to the persistence of chronic low-grade inflammation by activating ferroptosis, promoting aging-related renal fibrosis [70]. Therefore, we further explore the ferroptosis characteristics of macrophages in LFH. The GSVA analysis on LFH specimens revealed that SPP1^+^ Mac strengthened the pathways related to iron and lipid metabolism, suggesting that ferroptosis regulation may serve as an important component of the crosstalk between SPP1^+^ Mac and fibroblasts during LFH progression.

**Fig. 9:**
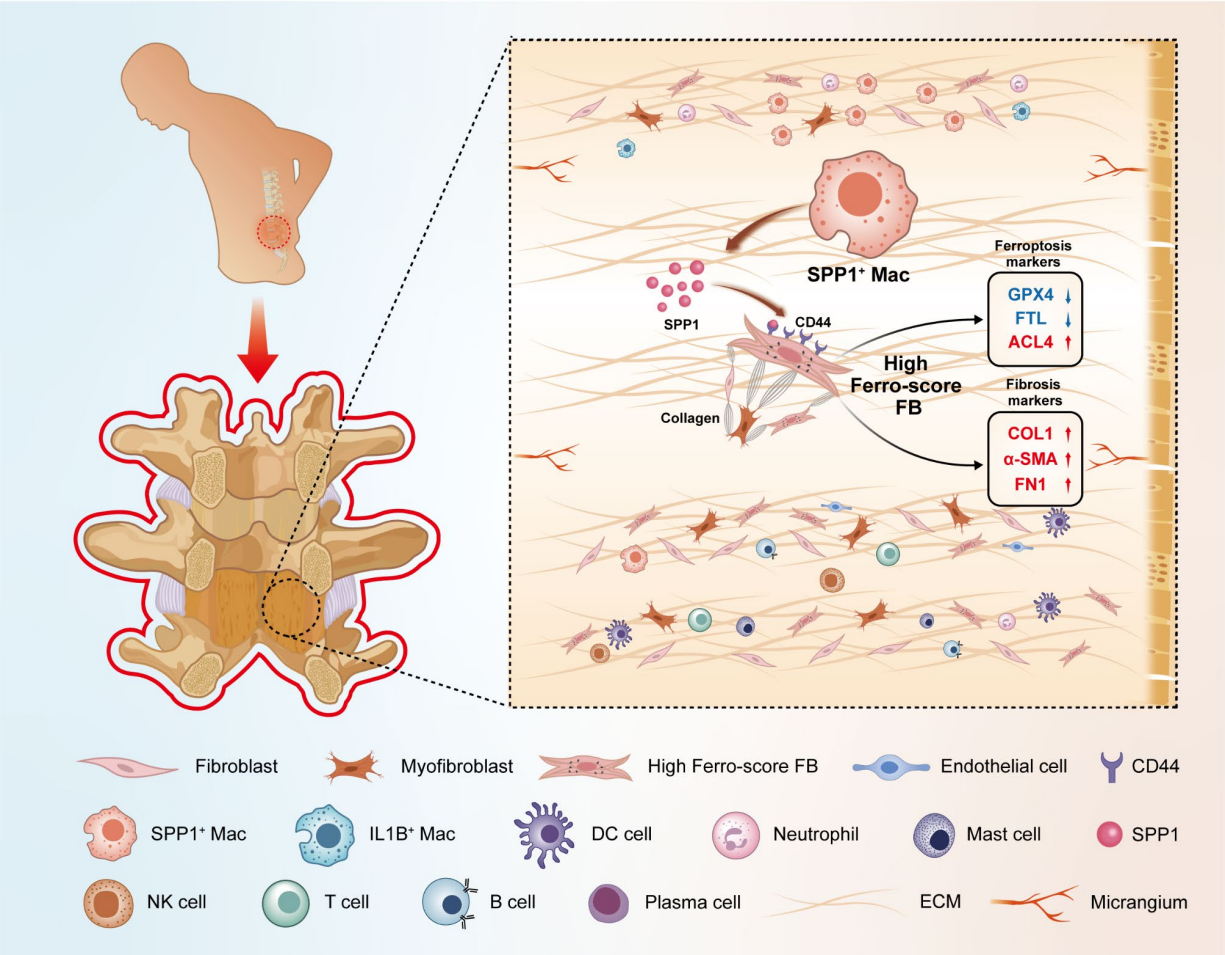
Schematic illustration of the fibrotic microenvironment during LFH degeneration. SPP1^+^ Mac regulate ferroptosis in fibroblasts through the SPP1-CD44 axis, thereby driving fibrosis in LFH

Given these findings, to better understand the role of immune cells in the fibrotic microenvironment of LF, we established multiple signaling pathways and cellular communication networks involved in the LFH disease progression. This analysis revealed the dysregulated crosstalk between High Ferro-score FB and macrophages in degenerative LF tissue. Numerous studies have indicated that fibroblasts and macrophages are extensively distributed throughout the body. They are not only spatially close to each other but also maintain mutual interactions and perform functions through cellular circuits mediated by exchanging growth factors [71, 72]. The disrupted crosstalk between fibroblasts and macrophages is regarded as a key driving force in fibrotic diseases [15]. Activated macrophages drive the activation, migration, and proliferation of fibroblasts by secreting factors such as TGF-β1, PDGF and AREG [72]. A new finding from our CellChat analysis is that SPP1^+^ Mac are the exclusive senders of the SPP1 signal in LFH, which has not been reported in previous studies. Both IHC and single-cell data demonstrated that the expression levels of SPP1 and its receptor CD44 are significantly upregulated in hypertrophic LF tissue compared to non-hypertrophic LF tissue. Furthermore, our analysis of L-R pairs revealed that SPP1^+^ Mac play a pivotal role in regulating High Ferro-score FB in LFH. The schematic illustration of the fibrotic microenvironment shows the cellular changes and the interaction between High Ferro-score FB and SPP1^+^ Mac during LFH degeneration (Fig. 9). Recently, it has been reported that the interaction between SPP1^+^ Mac and FAP^+^ fibroblasts may promote the formation of demyelinated regions in the tumor microenvironment by remodeling the ECM [73]. Some studies indicated that in perivascular adipose tissue, SPP1^+^ Mac can promote the migration and proliferation of fibrogenic progenitors through OPN-CD44/integrin interactions, thereby exacerbating fibrosis progression [74]. Our multiplex immunofluorescence results showed that the SPP1-CD44 colocalized regions in degenerative LF tissue are accompanied by COL1 deposition and a reduction in the ferroptosis-resisting gene *FTL*. This further confirms that SPP1^+^ Mac contribute to LFH fibrosis by regulating ferroptosis in fibroblasts through the SPP1-CD44 axis. Therefore, the SPP1 signaling pathway may serve as a potential target for treating LFH, warranting further investigation into its function.

Despite the significant findings, our study acknowledges certain limitations. Firstly, there were constraints in the feature selection of LF samples. During the study design phase, we categorized the samples into non-LFH and LFH groups without establishing a more detailed single-cell atlas based on disease severity. Moreover, we could not rule out age as a confounding factor in LFH degeneration. To minimize the impact of confounding factors on the accuracy of experimental results, the inclusion criteria were strictly controlled during sample collection and combined analyses were performed with publicly available datasets. However, given that LFH is clinically more prevalent in the elderly and a significant age disparity existed between the two groups, the influence of aging on the findings cannot be entirely excluded. In future research, we plan to include normal LF samples from elderly individuals for scRNA-seq analysis to mitigate the effect of age-related factors on experimental outcomes. Another limitation is that the mechanistic research is not yet thorough enough. The specific molecular mechanisms by which SPP1^+^ Mac regulate ferroptosis in fibroblasts, and how ferroptosis contributes to LF fibrosis have not been fully elucidated. A primary technical challenge lies in the isolation of SPP1^+^ Mac from LF tissues of human and mice. While recent studies have successfully isolated SPP1^+^ Mac from human flexor tendons [53], analogous methodologies for mice LF tissues remain underdeveloped, with few success reported to date. This gap may be addressed through the future construction of an effective and non-invasive rat LFH model. Additionally, the lack of widely validated and specific inhibitors or blockers targeting SPP1^+^ Mac currently hinders the in vivo validation of the promoting effect of SPP1^+^ Mac in LFH pathogenesis. We noticed that Liu et al. successfully reduced the content of SPP1^+^ Mac in the tumor immune barrier of hepatocellular carcinoma mice by injecting anti-SPP1 antibody [75]. Furthermore, several researchers have successfully constructed *Spp1* myeloid-specific knockout mice [76]. These methods may be applied in future studies to further clarify the specific role of SPP1^+^ Mac during LFH progression.

## Conclusions

In conclusion, our study conducted multi-omics analyses using both scRNA-seq and bulk RNA-seq to map the single-cell atlas of normal and degenerated human LF tissue. We innovatively revealed and validated the molecular changes in ferroptosis and SPP1^+^ Mac during the LFH fibrosis process. More importantly, these findings reveal the complex fibrotic microenvironment landscape and the novel macrophage-ferroptosis regulatory mechanism in which LFH initiates and progresses pathophysiologically. Despite certain limitations, our findings significantly enhance the current understanding of the pathogenesis of LFH. We believe that future exploration of ferroptosis mechanisms and the roles of specific macrophage subpopulations may hold significant value for the treatment of LFH and the development of targeted therapies.

## Electronic supplementary material

Below is the link to the electronic supplementary material.

Additional file 1: table S1. LF specimens for each experiment used in this study.

Additional file 2: table S2. Ferroptosis-promoting genes and Ferroptosis-resisting genes.

Additional file 3: table S3. Clinical information of 26 donors involved in this study.

Additional file 4: table S4. Marker genes of 21 clusters.

Additional file 5: table S5. MR table heterogeneity and MR table pleiotropy.

Additional file 6: table S6. GSEA pathway enrichment results of bulkRNA-seq.

Additional file 7: fig. S1. The UMAP visualization of 21,301 cells retained in human LF tissues after quality control of scRNA-seq data shows 21 clusters. Fig. S2. The ferrous ion (Fe^2+^) level of LF tissues in non-LFH group and LFH group. Fig. S3. Heatmap of GSVA score for each fibroblast subset. Red indicates high score, blue indicates low score. Fig. S4. The histogram of the proportion of fibroblast subsets in non-LFH group and LFH group. Fig. S5. The results of EVG and Masson staining in Control group and BS group. Fig. S6. PCA results before and after the merging of two datasets GSE113212 and GSEzzm. Fig. S7. The standard workflow for Bayesian Prism deconvolution analysis.

## Data Availability

All data generated and analyzed in the study can be obtained by contacting the corresponding author on reasonable request.

## Abbreviations

LFH: Ligamentum flavum hypertrophy
LSS: Lumbar spinal stenosis
LF: Ligamentum flavum
Bulk RNA-seq: Bulk RNA sequencing
MR: Mendelian randomization
MPs: Mononuclear phagocytes
SPP1^+^ Mac: SPP1^+^ macrophages
PCD: Programmed cell death
IHC: Immunohistochemistry
BS: Bipedal standing
MRI: Magnetic resonance imaging
UMAP: Uniform manifold approximation and projection
DEGs: Differentially expressed genes
CMCs: Cycling myeloid cells
GMPs: Granulocyte monocyte progenitors
EVG: Elastica van gieson
TEM: Transmission electron microscopy
ROS: Reactive oxygen species
GWAS: Genome-wide association study
SCS: Spinal canal stenosis
IVW: Inverse-variance weighted
SNPs: Single nucleotide polymorphisms
CI: Confidence interval
OR: Odds ratio
GO: Gene Ontology
DamFB: Damaged Fibroblast
HomFB: Homeostasis-associated Fibroblast
StrFB: Structural Fibroblast
ChoFB: Chondrogenic Fibroblast
InfFB: Inflammation-related Fibroblast
RepFB: Repair-related Fibroblast
MyoFB: Myofibroblast
ImmFB: Immune-related Fibroblast
GSVA: Gene Set Variation Analysis
High Ferro-score FB: Fibroblast with a high ferroptosis score
Low Ferro-score FB: Fibroblast with a low ferroptosis score
No significant FB: Fibroblast with a no significant ferroptosis score
GEO: Gene Expression Omnibus
PCA: Principal component analysis
GSEA: Gene Set Enrichment Analysis
IL1B^+^ Mac: IL1B^+^ macrophages
NES: Normalized enrichment score
FC: Fold change
cMon: Classical monocyte
MPC: Megakaryocyte progenitor cell
pDC: Plasmacytoid dendritic cell
cDC2: Conventional dendritic cell type 2
cDC3: Conventional dendritic cell type 3
Mø: Macrophages
M1: Classically activated macrophages
M2: Alternatively activated macrophages
L-R: Ligand-receptor
LDH: Lumbar disc herniation
TSA: Tyramide signal amplification
α-SMA: α-Smooth Muscle Actin
ACTA2: Actin Alpha 2
GPX4: Glutathione Peroxidase 4
FTL: Ferritin Light Chain
ACSL4: Acyl-CoA Synthetase Long Chain Family Member 4
COL1A2: Collagen Type I Alpha 2 Chain
FN1: Fibronectin 1
COL1: Collagen I
GAPDH: Glyceraldehyde-3-Phosphate Dehydrogenase
NAMPT: Nicotinamide phosphoribosyltransferase
NAD+: Nicotinamide adenine dinucleotide
HRP: Horseradish peroxidase
OPN: Osteopontin
DCN: Decorin
MYLK: Myosin Light Chain Kinase
VWF: Von Willebrand Factor
EMCN: Endomucin
KRT1: Keratin 1
KRT6A: Keratin 6A
HBD: Hemoglobin Subunit Delta
AHSP: Alpha Hemoglobin Stabilizing Protein
MKI67: Marker of Proliferation Ki-67
TOP2A: DNA Topoisomerase II Alpha
AZU1: Azurocidin 1
ELANE: Elastase, Neutrophil Expressed
C1QA: Complement C1q A Chain
CSF1R: Colony Stimulating Factor 1 Receptor
CSF3R: Colony Stimulating Factor 3 Receptor
IRF7: Interferon Regulatory Factor 7
CLIC3: Chloride Intracellular Channel 3
IL7R: Interleukin 7 Receptor
GNLY: Granulysin
NKG7: Natural Killer Cell Granule Protein 7
MS4A1: Membrane Spanning 4-Domains A1
IGHG1: Immunoglobulin Heavy Constant Gamma 1
IGHG4: Immunoglobulin Heavy Constant Gamma 4
TPSAB1: Tryptase Alpha/Beta 1
TPSB2: Tryptase Beta 2
NEAT1: Nuclear Paraspeckle Assembly Transcript 1
MALAT1: Metastasis Associated Lung Adenocarcinoma Transcript 1
XIST: X Inactive Specific Transcript
MT1G: Metallothionein 1G
MT1X: Metallothionein 1X
COL3A1: Collagen Type III Alpha 1 Chain
POSTN: Periostin
ACAN: Aggrecan
HAPLN1: Hyaluronan and Proteoglycan Link Protein 1
SOX9: SRY-Box Transcription Factor 9
CHAD: Chondroadherin
C1R: Complement C1r
CXCL12: C-X-C Motif Chemokine Ligand 12
CXCL2: C-X-C Motif Chemokine Ligand 2
MFAP4: Microfibril Associated Protein 4
FBLN1: Fibulin 1
TPPP3: Tubulin Polymerization Promoting Protein Family Member 3
PRG4: Proteoglycan 4
ITGB8: Integrin Subunit Beta 8
NTN4: Netrin 4
RGS5: Regulator of G Protein Signaling 5
ITGA2B: Integrin Subunit Alpha 2B
IRF8: Interferon Regulatory Factor 8
LILRA4: Leukocyte Immunoglobulin Like Receptor A4
CLEC4C: C-Type Lectin Domain Family 4 Member C
FCER1A: Fc Epsilon Receptor Ia
CD1C: CD1c Molecule
CD1E: CD1e Molecule
CCR7: C-C Motif Chemokine Receptor 7
MAP1A: Microtubule Associated Protein 1A
MRC1: Mannose Receptor C-Type 1
TNF: Tumor Necrosis Factor
FAP: Fibroblast Activation Protein Alpha
TFRC: Transferrin Receptor
TGF-β1: Transforming Growth Factor Beta 1
PDGF: Platelet Derived Growth Factor
AREG: Amphiregulin
