# Supplementary material for: Single-cell multi-omics analysis identifies SPP1+ macrophages as key drivers of ferroptosis-mediated fibrosis in ligamentum flavum hypertrophy
Source: Biomark Res. 2025 Feb 25;13:33. doi: 10.1186/s40364-025-00746-6 (PMC11863437; doi:10.1186/s40364-025-00746-6)
Supplement: Supplementary file 2 — Additional file 2: table S2. Ferroptosis-promoting genes and Ferroptosis-resisting genes. [file 40364_2025_746_MOESM2_ESM.docx]

**Table S3. Clinical information of 26 donors involved in this study**

| **Variable** | **Non-LFH Group**  **(n = 13)** | **LFH Group**  **(n = 13)** | ***p-*value** |
| --- | --- | --- | --- |
| Age (years) | 38.460±14.290 | 65.770±5.862 | < 0.001 |
| Sex assigned at birth (male:female) | 8:5 | 5:8 | – |
| LF thickness (mm) | 2.911±0.462 | 5.609±0.634 | < 0.001 |
| Lumbar level | L4/5 | L4/5 | – |

Independent sample *t*-test; data are presented as the mean ± SD; *p* < 0.05 is considered to be statistically significant. LF, ligamentum flavum; LFH, Ligamentum flavum hypertrophy.
