# Supplementary material for: Single-cell multi-omics analysis identifies SPP1+ macrophages as key drivers of ferroptosis-mediated fibrosis in ligamentum flavum hypertrophy
Source: Biomark Res. 2025 Feb 25;13:33. doi: 10.1186/s40364-025-00746-6 (PMC11863437; doi:10.1186/s40364-025-00746-6)
Supplement: Supplementary file 4 — Additional file 4: table S4. Marker genes of 21 clusters. [file 40364_2025_746_MOESM4_ESM.docx]

**Table S5. MR table heterogeneity and MR table pleiotropy**

MR table heterogeneity

| **id.exposure** | **id.outcome** | **outcome** | **exposure** | **method** | **Q** | **Q_df** | **Q_pval** |
| --- | --- | --- | --- | --- | --- | --- | --- |
| finn-b-E4_IRON_MET | ebi-a-GCST90018922 | Spinal canal stenosis | Disorders of iron metabolism | MR Egger | 0.020 | 1 | 0.886 |
| finn-b-E4_IRON_MET | ebi-a-GCST90018922 | Spinal canal stenosis | Disorders of iron metabolism | Inverse variance weighted | 0.864 | 2 | 0.649 |

All values remain three decimal places after the decimal point.

MR table pleiotropy

| **id.exposure** | **id.outcome** | **outcome** | **exposure** | **egger_intercept** | **se** | **pval** |
| --- | --- | --- | --- | --- | --- | --- |
| finn-b-E4_IRON_MET | ebi-a-GCST90018922 | Spinal canal stenosis | Disorders of iron metabolism | 0.126 | 0.138 | 0.527 |

All values remain three decimal places after the decimal point.
