# Supplementary material for: Single-cell multi-omics analysis identifies SPP1+ macrophages as key drivers of ferroptosis-mediated fibrosis in ligamentum flavum hypertrophy
Source: Biomark Res. 2025 Feb 25;13:33. doi: 10.1186/s40364-025-00746-6 (PMC11863437; doi:10.1186/s40364-025-00746-6)
Supplement: Supplementary file 6 — Additional file 6: table S6. GSEA pathway enrichment results of bulkRNA-seq. [file 40364_2025_746_MOESM6_ESM.docx]

**Table S1. LF specimens for each experiment used in this study**

| **Total specimens** | **Storage** | **Experiments** |
| --- | --- | --- |
| 10 LF specimens ( Non-LFH : LFH = 5 : 5 ) were equally cut into 20 LF specimens ( Non-LFH : LFH = 10 : 10 ) for experimental verification. | 4 LF specimens were stored in 2.5% glutaraldehyde. | Transmission electron microscopy  (Non-LFH : LFH = 2:2) |
|  | 10 LF specimens were stored in 4% paraformaldehyde. | Histological staining assays  (Masson, EVG, IHC, multiplex immunofluorescent)  (Non-LFH : LFH = 5:5) |
|  | 6 LF specimens were stored in liquid nitrogen.  ( These specimens were divided into two parts for different experiments ) | Western blotting  (Non-LFH : LFH = 3:3) |
|  |  | Detection of ferrous ions  (Non-LFH : LFH = 3:3) |
| 16 LF specimens ( Non-LFH : LFH = 8 : 8 ) were used for sequencing. | 10 LF specimens were used to prepare single-cell suspensions. | Single-cell RNA sequencing  (Non-LFH : LFH = 5:5) |
|  | 6 LF specimens were stored in liquid nitrogen. | Transcriptome sequencing  (Non-LFH : LFH = 3:3) |

Abbreviations: LFH, ligamentum flavum hypertrophy, LF, ligamentum flavum, IHC, immunohistochemistry. EVG, Elastica van gieson.
