# Supplementary material for: Single-cell multi-omics analysis identifies SPP1+ macrophages as key drivers of ferroptosis-mediated fibrosis in ligamentum flavum hypertrophy
Source: Biomark Res. 2025 Feb 25;13:33. doi: 10.1186/s40364-025-00746-6 (PMC11863437; doi:10.1186/s40364-025-00746-6)
Supplement: Supplementary file 7 — Additional file 7: fig. S1. The UMAP visualization of 21,301 cells retained in human LF tissues after quality control of scRNA-seq data shows 21 clusters. Fig. S2. The ferrous ion (Fe2+) level of LF tissues in non-LFH group and LFH group. Fig. S3. Heatmap of GSVA score for each fibroblast subset. Red indicates high score, blue indicates low score. Fig. S4. The histogram of the proportion of fibroblast subsets in non-LFH group and LFH group. Fig. S5. The results of EVG and Masson staining in Control group and BS group. Fig. S6. PCA results before and after the merging of two datasets GSE113212 and GSEzzm. Fig. S7. The standard workflow for Bayesian Prism deconvolution analysis. [file 40364_2025_746_MOESM7_ESM.docx]

**Title**: Single-cell Multi-omics Analysis Identifies SPP1^+^ Macrophages as Key Drivers of Ferroptosis-Mediated Fibrosis in Ligamentum Flavum Hypertrophy


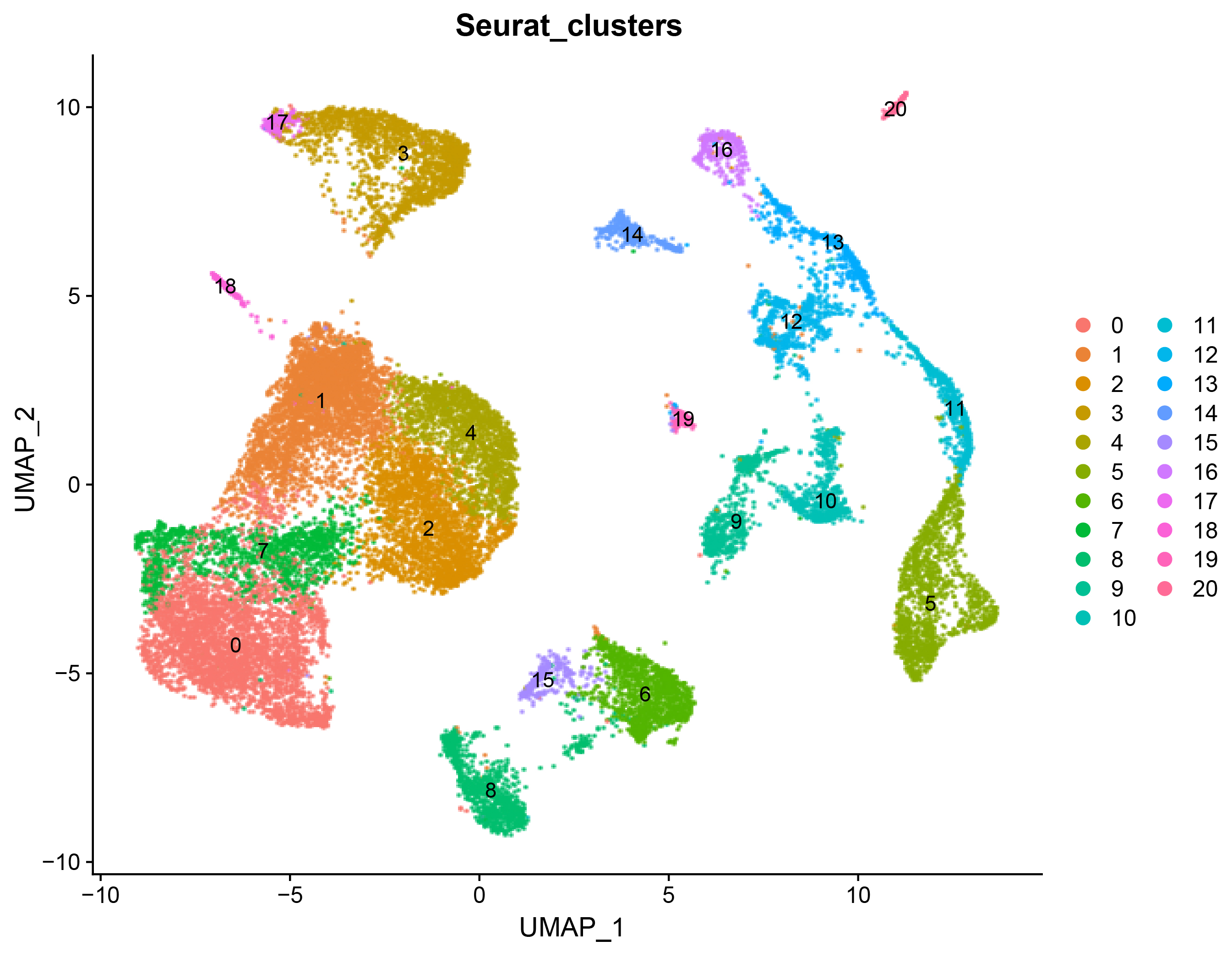


**Fig. S1.** **The UMAP visualization of 21,301 cells retained in human LF tissues after quality control of scRNA-seq data shows 21 clusters.** Abbreviations: ScRNA-seq, single cell RNA sequencing. LF, ligamentum flavum. UMAP, uniform manifold approximation and projection.





**Fig. S2. The ferrous ions (Fe^2+^) level of LF tissues in non-LFH group and LFH group (n=3).** Data quantification results are presented as mean ± SD, with ****p* < 0.001. Abbreviations: LFH, ligamentum flavum hypertrophy.


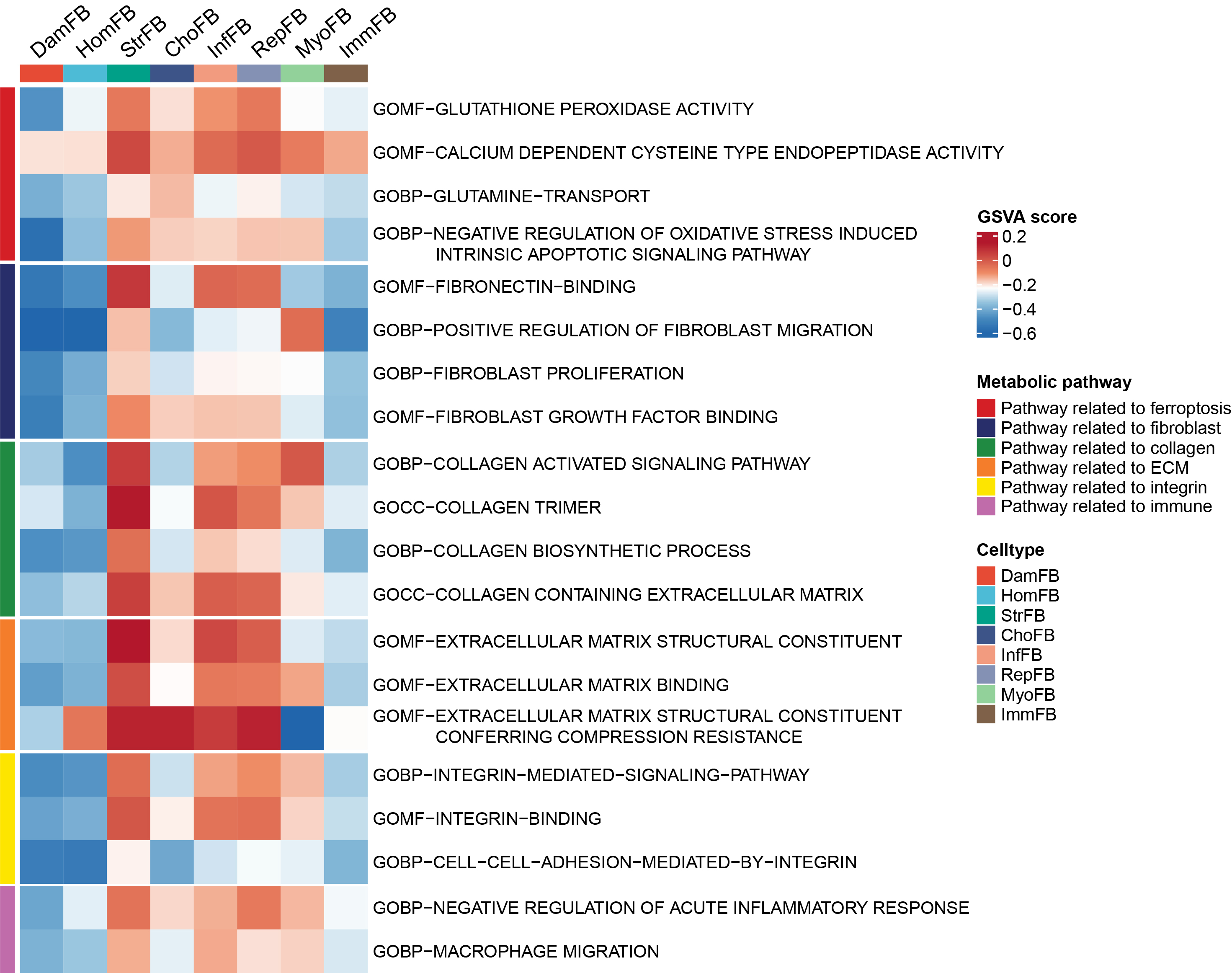


**Fig. S3. Heatmap of GSVA score for each fibroblast subset. Red indicates high score, blue indicates low score.** Abbreviations: GSVA, gene set variation analysis. DamFB, damaged fibroblast. HomFB, homeostasis-associated fibroblast. StrFB, structural fibroblast. ChoFB, chondrogenic fibroblast. InfFB, inflammation-related fibroblast. RepFB, repair-related fibroblast. MyoFB, myofibroblast. ImmFB, immune-related fibroblast. ECM, Extracellular matrix GO, Gene Ontology


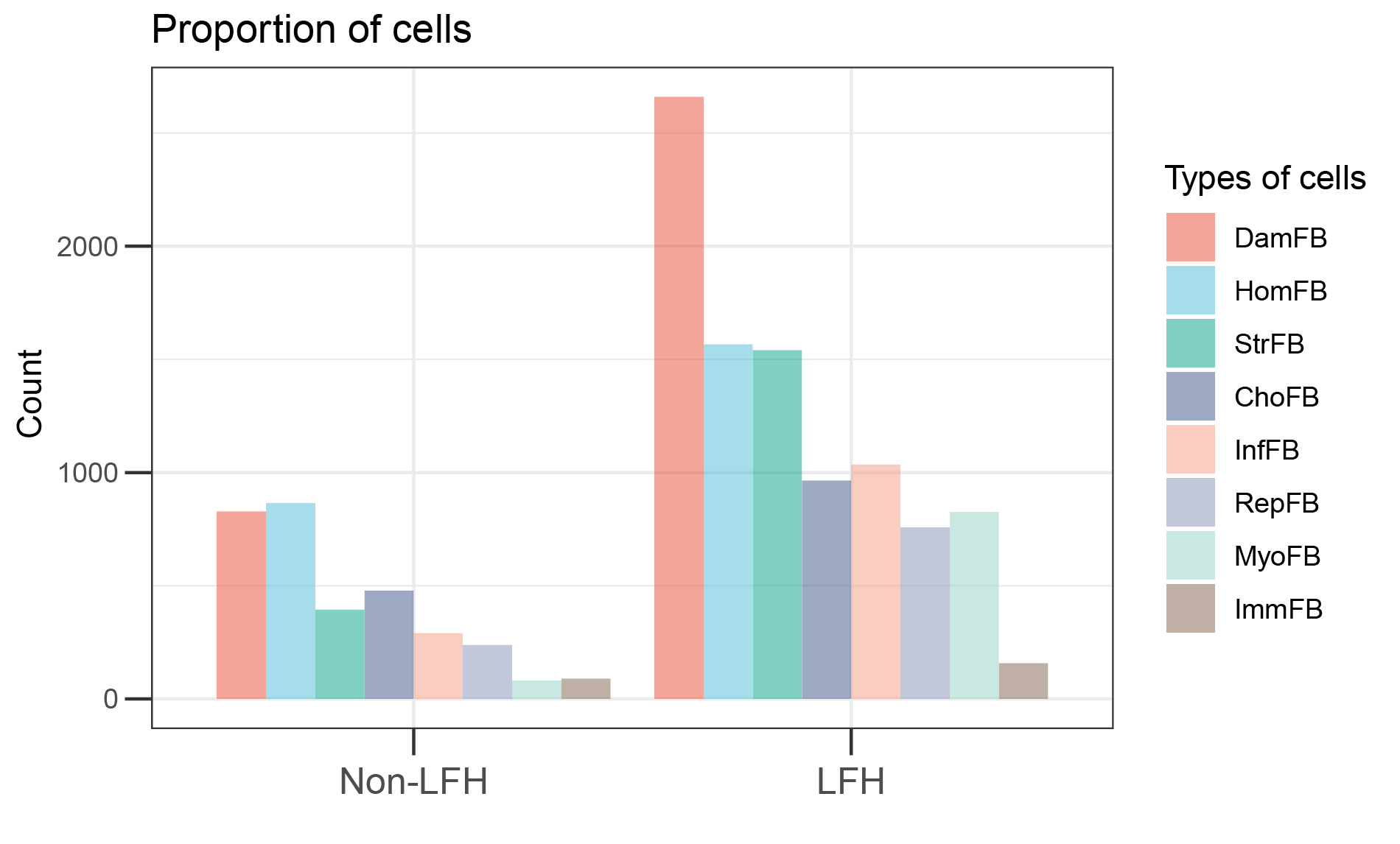


**Fig. S4. The histogram of the proportion of fibroblast subsets in non-LFH group and LFH group.** Abbreviations: LFH, ligamentum flavum hypertrophy. DamFB, damaged fibroblast. HomFB, homeostasis-associated fibroblast. StrFB, structural fibroblast. ChoFB, chondrogenic fibroblast. InfFB, inflammation-related fibroblast. RepFB, repair-related fibroblast. MyoFB, myofibroblast. ImmFB, immune-related fibroblast.


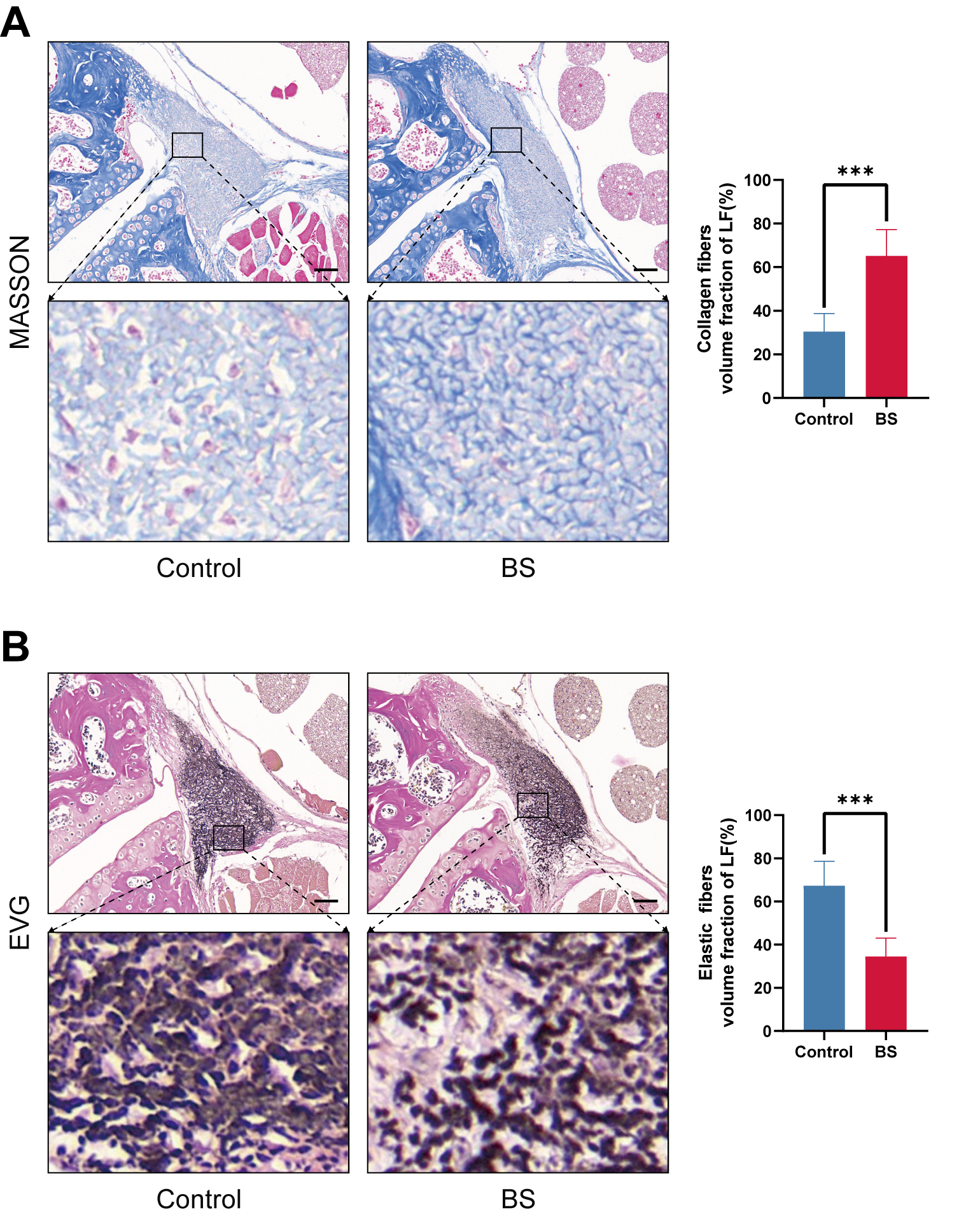


**Fig. S5. The results of EVG and Masson staining in Control group and BS group.** (A) In Masson staining, collagen fibers were stained blue and elastic fibers were stained red. (B) In EVG staining, collagen fibers were stained red and elastic fibers were stained black. The accompanying bar graph presents a quantitative analysis of the volume fractions (%) of elastic and collagen fibers in LF tissue of mice. Scale bar, 50μm. Data quantification results are presented on the right as mean ± SD, with ****p* < 0.001. Abbreviations: EVG, Elastica van gieson. BS, bipedal standing. LF, ligamentum flavum.


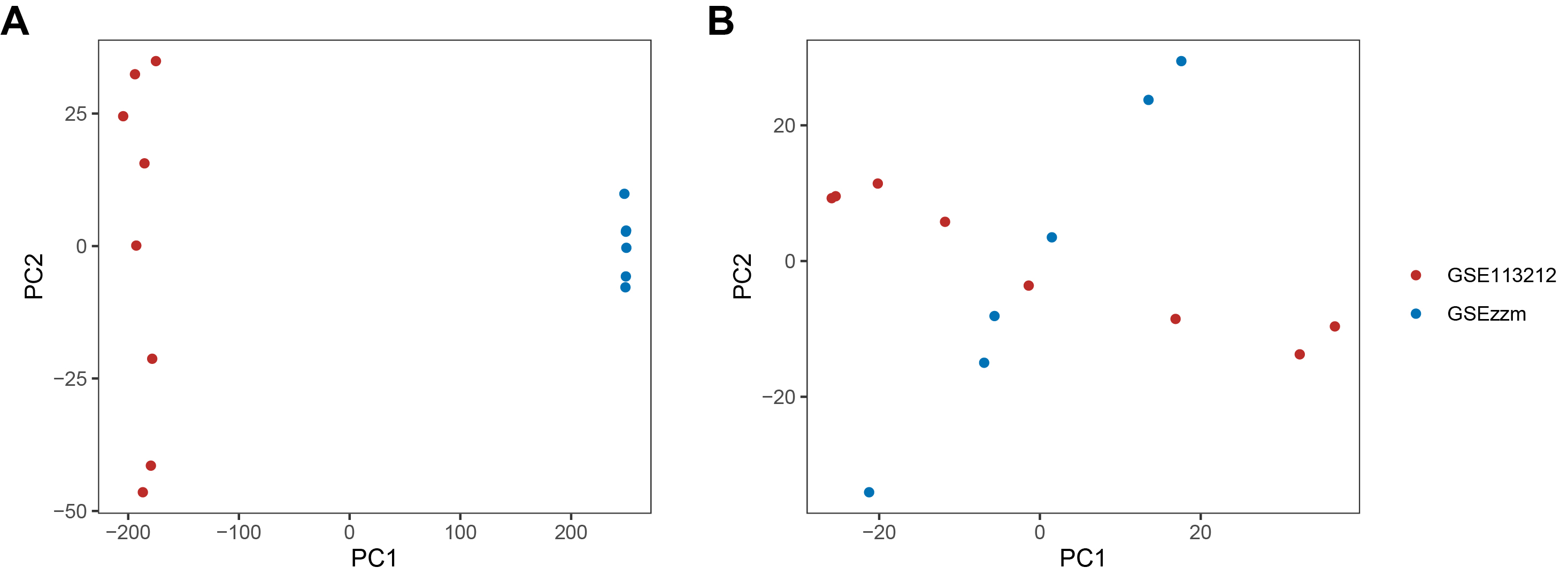


**Fig. S6. PCA results before and after the merging of two datasets GSE113212 and GSEzzm.** (A) before merging. (B) after merging. Abbreviations: PCA, principal component analysis.


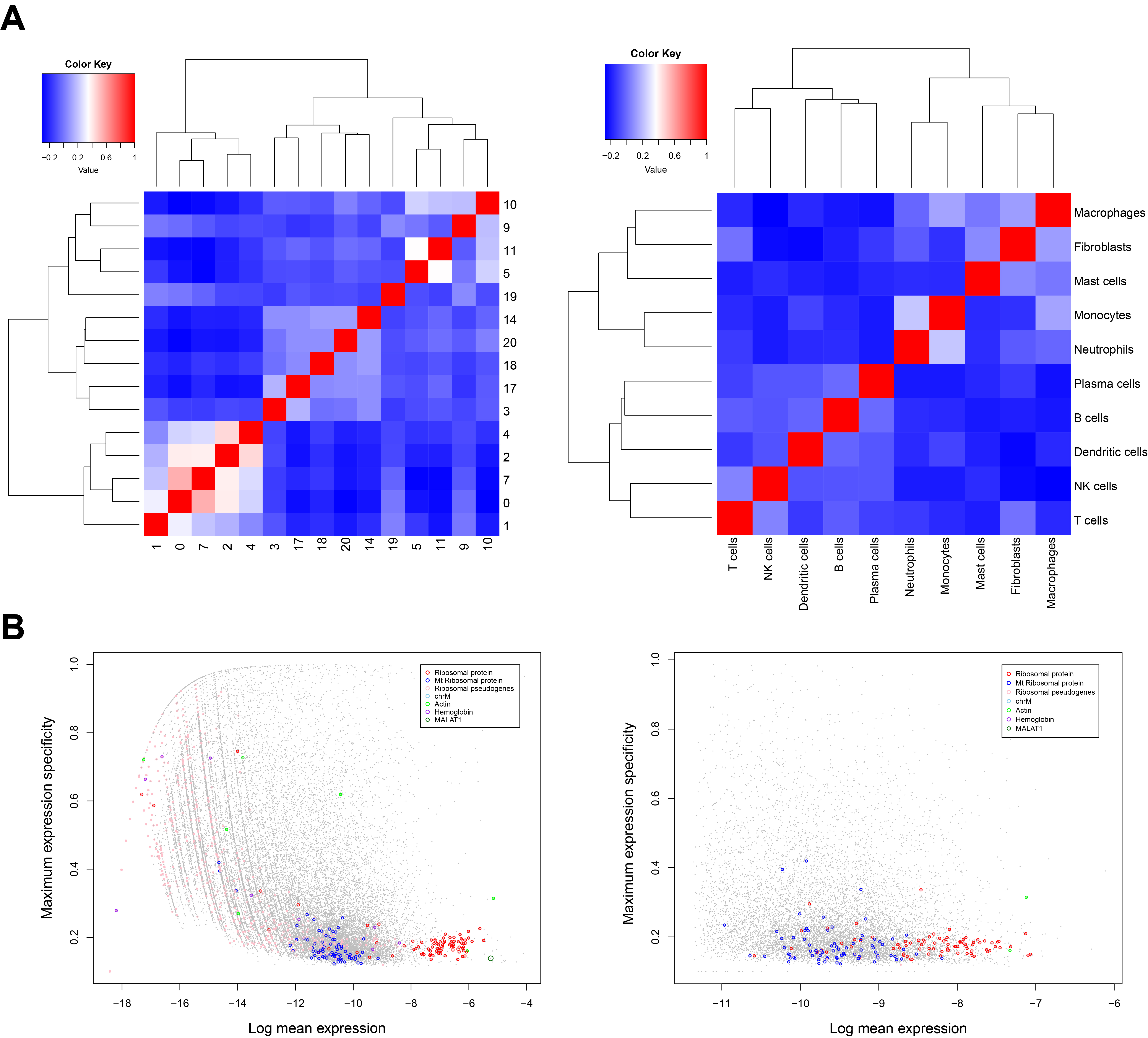


**Fig. S7. The standard workflow for Bayesian Prism deconvolution analysis.** (A) The correlation analysis results of cell type and cell state of scRNA-seq, the left is the cell state, and the right is the cell type. (B) The outlier gene map of BayesPrism deconvolution analysis. The left is the outlier gene map of single cell data, and the right is the outlier gene map of Bulk RNA-seq. Abbreviations: ScRNA-seq, single-cell RNA sequencing. Bulk RNA-seq, bulk RNA sequencing.
